# Agent-based modelling and parameter sensitivity analysis with a finite-element method for skin contraction

**DOI:** 10.1007/s10237-020-01354-z

**Published:** 2020-07-04

**Authors:** Qiyao Peng, Fred Vermolen

**Affiliations:** 1 Delft Institute of Applied Mathematics, Delft University of Technology, Van Mourik Broekmanweg 6, 2628 XE Delft, The Netherlands; 2 Computational Mathematics Group (CMAT), Division of Mathematics and Statistics, Faculty of Sciences, University of Hasselt, Agoralaan building D, 3590 Diepenbeek, Belgium

**Keywords:** Wound healing, Wound contractions, Monte Carlo simulations, Finite-element method, Agent-based modelling

## Abstract

In this paper, we extend the model of wound healing by Boon et al. (J Biomech 49(8):1388–1401, 2016). In addition to explaining the model explicitly regarding every component, namely cells, signalling molecules and tissue bundles, we categorized fibroblasts as regular fibroblasts and myofibroblasts. We do so since it is widely documented that myofibroblasts play a significant role during wound healing and skin contraction and that they are the main phenotype of cells that is responsible for the permanent deformations. Furthermore, we carried out some sensitivity tests of the model by modifying certain parameter values, and we observe that the model shows some consistency with several biological phenomena. Using Monte Carlo simulations, we found that there is a significant strong positive correlation between the final wound area and the minimal wound area. The high correlation between the wound area after 4 days and the final/minimal wound area makes it possible for physicians to predict the most probable time evolution of the wound of the patient. However, the collagen density ratio at the time when the wound area reaches its equilibrium and minimum, cannot indicate the degree of wound contractions, whereas at the 4th day post-wounding, when the collagen is accumulating from null, there is a strong negative correlation between the area and the collagen density ratio. Further, under the circumstances that we modelled, the probability that patients will end up with 5% contraction is about 0.627.

## Introduction

Wound healing is the spontaneous process of the skin to cure itself after an injury. It is a complex cascade of cellular events which contribute to resurfacing, reconstitution and restoration of the tensile strength of injured skin.

Roughly speaking, skin consists of three layers: the epidermis, the dermis and the hypodermis. Superficial wounds will heal without any problem, since the trauma only stays on the epidermis. However, if it is a severe, deeper injury at the dermis, which causes a significant loss of soft tissue, then the dermal wounds may lead to various pathological problems. Therefore, it is vital for the skin that (secondary) deep wound healing proceeds speedy and effective. During secondary healing, the formation of a blood clot, the regeneration of collagen (in extracellular matrix) and re-vascularisation take place (Enoch and Leaper 2008). In most cases of serious skin trauma, excessive healing reactions, such as the development of wound contractures, known as excessive and pathological contractions, or hypertrophic scars, takes place.

Contractions are caused by interactions between cells and extracellular matrix (ECM), that is, (myo)fibroblasts exert pulling forces on their immediate surroundings. By this contractile mechanism, large, severe wounds in human skin reduce by 5–10% of its original size. Furthermore, the polymeric structure and orientation of regenerated collagen will be different from embryonic, undamaged skin (Enoch and Leaper 2008).

**Fig. 1:**
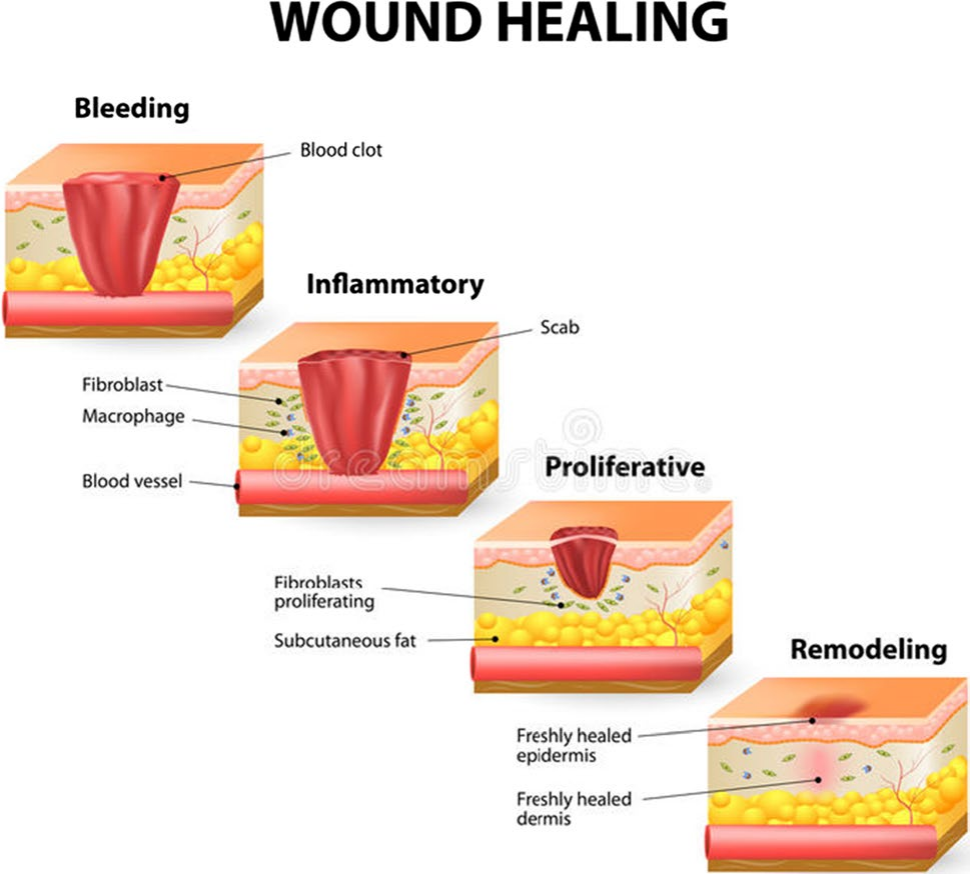
Four partially overlapping stages of wound healing (Advisor 2016)

Wound healing entails four partially overlapping phases: hemostasis, inflammation, proliferation and maturation/remodelling (Enoch and Leaper 2008); see Fig. 1. During hemostasis, mainly platelets are responsible for clot formation so that the clot can prevent more blood loss and also provides the provisional scaffold for cell migration towards the wound (Cumming et al. 2009). Subsequently, the inflammatory cells (white blood cells) are attracted to the wound to remove the bacteria and debris in inflammatory phase. When it comes to the next proliferative phase, different entities like vessels, fibrin and granulation tissues, etc., start being regenerated and the wound begins to contract. In the final remodelling stage, collagen forms tight cross-links, which increases the tensile strength of the scar. Note that the remodelling might take from months to years depending on the wound.

The four phases will be explained in more detail in the coming text, since the evolution of skin entails a complicated sequence of biological processes, we can only summarize the biological dynamics.

Immediately after injury, hemostasis starts, which is characterized by vasoconstriction—a process to stop bleeding by closing damaged blood vessels (Cumming et al. 2009; Rosińczuk et al. 2016). If the lining of the blood vessels is broken, then the nearby uninjured blood vessels will constrict to limit blood loss. As a result, a blood clot is developed to seal off the wound from its surroundings, so that the invasion of other hazardous contaminants and pathogens into the skin is prevented. In the meanwhile, the platelets are activated and aggregating, which leads to the clot formation. The activation facilitates platelets to degranulate and release chemotactic and growth factors into the extracellular space (Enoch and Leaper 2008).

Inflammation can be split into early (24–48 h) and late (48–72 h) phases. The main process during the inflammatory phase is characterized by high activity of the immune system. In other words, the inflammatory cells, including neureophils, macrophages and T lymphocytes will enter the wound to clean the damaged region, specifically by removing the bacteria and debris through phagocytosis (Haertel et al. 2014; Cumming et al. 2009). In the early stage of the phase, neurophils are the first immune cells arriving at the wound, followed by tissue macrophages which are originally from monocytes (Haertel et al. 2014). Macrophages are the most important cells in the later inflammatory phase stage since they are the original producers of cytokines, especially of transforming growth factor beta (TGF-beta), which stimulates the chemotaxis and proliferation of fibroblasts and smooth muscle cells (Enoch and Leaper 2008). At the final stage of the inflammatory phase, chemokines released by macrophages attract endothelial cells to the wound and stimulate angiogenesis, which is the formation of a blood vessel network. During this stage, T-lymphocytes, which mainly effect the cell-mediated response and release signalling molecules, also start moving to the wound area. Additionally, skin resident mast cells migrate to the wound area (Haertel et al. 2014).

The subsequent phase is proliferation, which will continue for 2–4 weeks after wounding, depending on the wound size (Enoch and Leaper 2008). It includes epithelialization, fibroplasia, angiogenesis and the development of granulation tissue. Redifferentiation of the keratinocytes in the neo-epidermis is activated before completing wound closure and leads to efficient reconstitution of the epidermal barrier (Haertel et al. 2014). After epithelialization, the injured dermis starts being repaired. Within 4 days, the clot will be broken by proteins like plasmins, of which the production is effected by the presence of tissue plasminogen activator (tPA). Subsequently, the clot is replaced by granulation tissue, which consists of cells and connective tissues (Cumming et al. 2009). During this process, fibroblasts are attracted to the wound area from the wound surroundings by a number of factors like platelet-derived growth factor (PDGF) and transforming growth factor-beta (TGF-beta) (Enoch and Leaper 2008). Once within the wound, fibroblasts sometimes differentiate into myofibroblasts, which pull the extracellular matrix with higher forces and cause deformation of the tissue (Haertel et al. 2014; Li and Wang 2011). In the meanwhile, fibroblasts release collagen to rebuild the ECM. However, the tensile and strength properties of the newly built collagen differ from these properties of uninjured skin. In the course of time, the tensile strength will increase as a result of a tight cross-link of the collagen molecules. Additionally, since the newly released collagen is deposited according to the direction of migration of the fibroblasts, the orientation of newly regenerated collagen is anisotropic. In summary, wound contraction takes place due to myofibroblasts exerting pulling forces on the surrounding extracellular matrix and results into the formation of (permanent) stresses and strain in and around the wound area. The amount of the contraction is related to the size, shape, depth and anatomical location of the wound, for instance, tissues with stronger laxity contract more compared to loose tissues, and square-shaped wounds contract more than circular ones (Enoch and Leaper 2008; O’Leary et al. 2008). Due to the occurrence of contraction, the exposed surface area of the wound decreases without the production of new wound-covering tissues. Notably, contraction must be distinguished from contracture, which is a pathological process of excessive contraction. Usually, contractures concur with disfunctioning and disabilities of the patients.

The late stage of new tissue formation overlaps with maturation/remodelling which can last several years. At last, the dermal tissue has a low cell density for both macrophages and fibroblasts (Enoch and Leaper 2008), and the tensile strength of new tissue will increase but it will never reach the level of undamaged tissue (Haertel et al. 2014; Enoch and Leaper 2008; Cumming et al. 2009). On the other hand, the main factors in the maturation/remodelling phase are collagen and cytokines. As mentioned earlier, the collagen molecules connect to other collagen molecules to strengthen the bundles. Cytokines are released from a variety of cells and bind to cell surface receptors to stimulate a cell response.

The aforementioned stages of wound healing partly overlap each other and intracellular communication drives the initiation and termination of the subsequent stages. The inflammatory phase is initiated by the platelets that originate from the blood vessels and form the blood clot. The platelets secrete PDGF, that is detected by the immune cells (white blood cells) in the blood circulatory system (for example, neurophils, monocytes, macrophages and T-lymphocytes). The immune cells transmigrate through the walls of the blood vessels to arrive at the wound site (Deuel et al. 1982), where they clear up debris and other pathogens through phagocytosis (Cumming et al. 2009; Haertel et al. 2014). These immune cells secrete the transforming growth factor TGF-beta. We bear in mind that the immune cells release several types of TGF-beta chemokines and that some types of these chemokines initiate the formation of a newly generated blood vessel network (also referred to as angiogenesis). Migration of the fibroblasts and immune cells takes place via various biological mechanisms. The most important biological mechanisms for cell migration are random walk, which takes place as a result of unpredictable inhomogeneities and anisotropies in the extracellular matrix of skin, chemo (hapto)taxis, which characterizes cellular migration in the direction of (or opposite to) the gradient of a chemical and tensotaxis, which represents cellular migration driven by mechanical cues.

For many of the biological mechanisms that take place during wound healing, mathematical models have been developed. These mathematical models range from all the phases that we listed so far. In general, there are two main types of models to simulate wound healing. One class of formalisms is formed by continuum models developed by Tranquillo and Murray (1992), among many others, in which all the behaviours of species are described by partial differential equations (PDEs). Cells are represented by densities rather than by entities of their own. The other class of models is hybrid framework based, in which the cells are treated as individual, discrete entities and the extracellular matrix (ECM) is treated as a continuous variable. Within this framework, we mainly incorporate PDEs to determine displacements of cells, concentration of signalling molecules and tissue bundles. Furthermore, the current paper focusses on the formation of contractions after serious injuries. For this reason, we will elucidate some of the biological mechanisms behind contraction formation.

In this manuscript, we will use the hybrid cell-based model developed by Vermolen and Gefen (2012) and improved by Boon et al. (2016). The innovations are the following: in this modelling study, a six-species model for the second and third phases of wound healing processes is selected, including multiple types of cells, cytokines and tissues; see Cumming et al. (2009) and Koppenol (2017). In the current text, we have improved the model by modelling the proliferation and differentiation of both fibroblasts and myofibroblasts by the use of stochastic sampling from exponential distributions. In the model, cells are taken as spherical individuals which become circles after projection in two spatial dimensions, cytokines and tissues are treated as continuous variables. Furthermore, we have done sensitivity tests to validate the model and Monte Carto simulations to assess the impact of uncertainty and parameter variation.

The manuscript is structured as follows. In Sect. 2, we present the biological assumptions and the semi-stochastic cell-based model. Section 3 treats the numerical results of the model from various aspects, like the positions of the cells, variations of the concentration of cytokines and strain energy of the wound, and the wound area changing over time, which is taken as an indicator of the contractions. Furthermore, the effect of applying different parameter values on wound healing is probed. The results from the Monte Carlo simulations are presented in Sect. 4. Finally, the conclusions and some remarks for the model are shown in Sect. 5.

## Mathematical models

### Biological assumptions

**Fig. 2:**
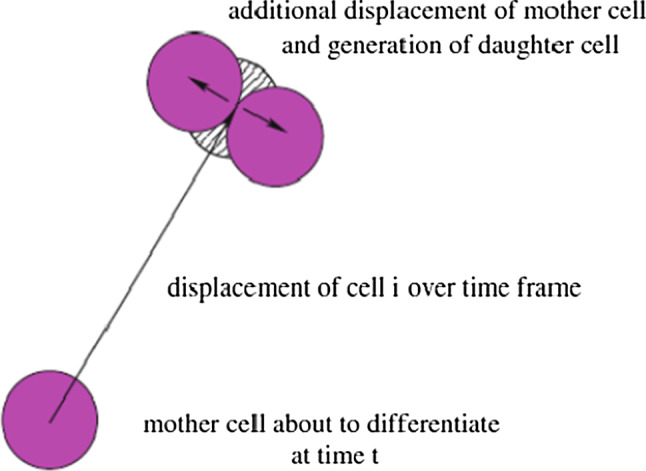
The process of cell division combined with the cell displacement Vermolen and Gefen (2012)

To regenerate new and healthy tissue in the wound, the importance of cell proliferation, migration and differentiation has been documented (Rosińczuk et al. 2016). However, the activities of viable cells are much more complex than they were observed in the laboratory. In other words, it is infeasible to depict and contain every aspect of cellular activity into the model. Therefore, to encode a mathematical model, we have to make some simplifications of biological system and until now we mainly work in two spatial dimensions. Under this circumstance, the domain we are working on is denoted by $\Omega \in \mathbb{R}^2$. Furthermore, we use the following assumptions for the cells—in this report, namely macrophages, regular fibroblasts and myofibroblasts:

- cells are in a circular shape with a fixed radius;
- distortion of cell geometry is not taken into consideration; however, cells are allowed overlap in a reasonable range;
- once two cells mechanically contact and even (partly) overlap, they will repel each other and exert a repulsion force which is in the opposite direction of the vector connecting the cells; additionally, the remote traction forces between any two cells are neglected;
- each cell is either viable or dead, and once a cell dies, it disappears immediately from the computational domain; we note that the finite-element method is applied over the entire domain, and hence the only thing that changes in the algorithm is the disappearance of a force or source as soon as a cell dies;
- when cell division occurs, the centre of the original cell moves randomly to the circumference and the new cell’s centre is on the opposite side of the circumference; the process is depicted in Fig. 2 Vermolen and Gefen (2012);
- each cell needs time to grow before it is allowed to differentiate or divide after its birth; the daughter cell needs more time than the mother cell (Di Talia et al. 2009), but cell death/apoptosis can always occur due to excessive mechanical forcing;
- cell division and death rates follow the exponential distribution; see Chen et al. (2017) for more details, of which the probability rate for these processes depends on the concentration of TGF-beta and strain energy density;
- in this model, the division of macrophages and myofibroblasts are neglected; hence, only regular fibroblasts are allowed to proliferate;
- each viable (myo)fibroblast exerts a force on the substrate where it is living on.

Note that in fact cells are not overlapping, but they collide against each other. Subsequently, they repel each other. We use a Hertz contact model to simulate repulsion against cells. This model is based on the indentation of a sphere, which we model by ‘overlapping’. Similar approaches by different authors can be found in for instance (Yamao et al. 2011). Some papers report about the quantification of cell forces; however, the experimental values of intracellular forces are characterized by very large uncertainties. The magnitude of intracellular forces, which effects the ‘over lapping region’ between cells does not effect our computed results in terms of contractile behaviour of burn injuries that much.

The solution of the partial differential equations that will be presented in this section is approximated by the use of the finite element method. The finite element method is applied over the entire domain of computation to approximate the solution of the equations for the concentrations and force balance. The individual cells act as sources for the regeneration of chemical agents and exert forces to the immediate surroundings. To this extent, the whole domain of computation is triangulated and linear basis functions are used. Since the cells exert forces to their surroundings, the region is deformed, which is incorporated into the finite element method. The finite-element method is implemented within the FEniCS package (Langtangen and Logg 2016). For completeness, we present the Galerkin formulations of the partial differential equations that we numerically solve.

Regarding the cell repelling force, a Hertz contact force is used to take cells as isotropic homogeneous elastic bodies, which are deformed if two cells repel each other. The difference between the sum of the radius of two cells and the distance between two cell centres is defined as overlapping distance (Chen et al. 2017; Van Liedekerke et al. 2018; Vermolen and Gefen 2012). This overlapping region is fictitious since in real situations cells do not overlap. The overlapping distance is used to quantify the indentation, which in turn, determines the repulsive force. We have used the Hertz contact force model for soft spheres.

We consider a two-dimensional computational domain that is filled with cells and substrate. Since the predominant mode of cellular migration is by chemotaxis, we only incorporate direct mechanical (repulsion) forces between cells that are in physical contact. For more details about modelling the traction forces using strain energy density, see Vermolen and Gefen (2012) and Dudaie et al. (2015).

Strain energy density is the mechanical energy per unit volume, and it quantifies the magnitude of the strain by which a cell will migrate.

The strain energy density from the repulsive force that is exerted by colliding cells is modelled by, see Vermolen and Gefen (2012):

1 $$\begin{aligned} M^{ij}\left( \varvec{r}_i\right) =\frac{1}{30}\frac{E_c}{\pi }\left( \frac{h_{ij}}{R}\right) ^{\frac{5}{2}}, \end{aligned}$$

where

$$\begin{aligned} h_{ij}=\max \left\{ 2R-\Vert \varvec{r}_i-\varvec{r}_j\Vert , 0\right\} , \end{aligned}$$

is known as the overlapping distance of two cells. We consider cell *i* as a cell that collides with other cells, then the total repulsion energy density is the sum of the repulsion energy densities which results from the cells contacting cell *i* mechanically. Suppose cells $j\in \left\{ i_1, \ldots , i_k\right\} \subseteq \left\{ 1, \ldots , n(t)\right\}$ contact mechanically with cell *i* at time *t*, then the total repulsion energy is

2 $$\begin{aligned} M^{mc}\left( \varvec{r}_i\right) =\sum _{j=i_1}^{i_k}M^{ij}(\varvec{r}_i),\quad \text{ for }\quad i\in \left\{ 1,\ldots ,n \right\} . \end{aligned}$$

Combining the results of traction and repulsion effect, as well as based on the cell assumptions, the total energy density is

3 $$\begin{aligned} \hat{M}(\varvec{r}_i)= {\left\{ \begin{array}{ll} 0, &{} \text{ if }\quad h_{ij}=0, \\ -M^{mc}\left( \varvec{r}_i\right) , &{} \text{ if }\quad h_{ij}>0. \end{array}\right. } \end{aligned}$$

According to Vermolen and Gefen (2012), the displacement of cell *i* over a time step $\Delta t$ is a linear combination of all the unit vectors connecting to the rest with the total strain energy density as the weight factor. Hereby, we use the unit vector connecting cell *i* to cell *j*

$$\begin{aligned} {\mathbf {e}}_{ij}=\frac{\varvec{r}_j-\varvec{r}_i}{\Vert \varvec{r}_j-\varvec{r}_i\Vert },\quad \text{ where }\quad i\ne j, \end{aligned}$$

and according to the biological assumption of no more traction force, the weight of the direction of displacement is given by

4 $$\begin{aligned} M_z(\varvec{r}_i)= {\left\{ \begin{array}{ll} 0, &{} \text{ if }\quad h_{ij}=0,\\ -M^{ij}(\varvec{r}_i), &{}\text{ if }\quad h_{ij}>0. \end{array}\right. } \end{aligned}$$

Then the overall moving direction of cell *i* is given by the following equation:

5 $$\begin{aligned} \varvec{z_i}=\sum _{j=1,j\ne i}^{n}M_z(\varvec{r}_i)\varvec{e}_{ij}= \sum _{j = i_1}^{i_k} M_z(\varvec{r}_i) \varvec{e}_{ij}. \end{aligned}$$

Then we normalize the vector again to obtain

$$\begin{aligned} \varvec{\hat{z}}_i=\frac{\varvec{z}_i}{\Vert \varvec{z}_i\Vert }. \end{aligned}$$

Based on the model of displacement of cell *i*, developed by Vermolen and Gefen (2012), the direction is determined by $\varvec{\hat{z}}_i$ and the magnitude of the displacement is proportional to the total energy density $\hat{M}(\varvec{r}_i)$.

To improve the computational efficiency, we use another method modelling the proliferation and apoptosis of cells, which is extended from the one used in Chen et al. (2017). Each viable cell has a certain possibility to proliferate or die due to its surroundings and its internal environment. In this manuscript, for proliferation and differentiation of regular fibroblasts, we consider the effects from the surroundings on each cell, namely the strain energy density due to forces exerted by other cells and the concentration of TGF-beta.

Following the approach in Vermolen and Gefen (2015), the probability of cell proliferation, differentiation and apoptosis follow a (memoryless) exponential distribution, of which the probability density function is defined by

$$\begin{aligned} f_{t_n}(\lambda ,t)=\lambda \exp \{-\lambda (t-t_n)\}, \end{aligned}$$

in the time interval $(t_, t_n+\Delta t)$. Here, $\Delta t$ is the timestep, which is fixed in this manuscript. Hence,

$$\begin{aligned} \mathbb{P}(t\in (t_n,t_n+\Delta t))&=\int _{t_n}^{t_n+\Delta t}\lambda \exp \{-\lambda (t-t_n)\}\mathrm{d}t\\&=1-\exp \{-\lambda \Delta t\}, \end{aligned}$$

and hence the probability is determined by the $\lambda \Delta t$. The division and apoptosis rates were used on the basis of earlier studies in Vermolen and Gefen (2015) and on the basis of Chen et al. (2017), where it has been specified how proliferation and death rates change with mechanical signals. These rates are incorporated in the exponential distribution, which we describe in Sect. 2.2. The actual values were based on ‘educated guesses’ and the sensitivity of the model. The probability rate is set according to the following relation with the strain energy density and the concentration of TGF-beta:

$$\begin{aligned} \begin{aligned} \lambda _d&=20\times c^2_{\mathrm{TGF}}(\varvec{x},t)\\&\quad +{\left\{ \begin{array}{ll} 2,&{}\text{ if }\quad \Vert \hat{M}(\varvec{r_i})\Vert <0.05\,\hbox{kg}/(\upmu \hbox{m}\,\hbox{h}^2),\\ 0,&{}\text{ otherwise }, \end{array}\right. } \end{aligned} \end{aligned}$$

where $c_{\mathrm{TGF}}(\varvec{x},t)$ is the concentration of TGF-beta at position $\varvec{x}$ and time *t*, and

$$\begin{aligned} \lambda _a= {\left\{ \begin{array}{ll} 10,&{}\text{ if }\quad \Vert \hat{M}(\varvec{r_i})\Vert \geqslant 0.07\,\hbox{kg}/(\upmu \hbox{m}\,\hbox{h}^2),\\ 0,&{}\text{ otherwise, } \end{array}\right. } \end{aligned}$$

for proliferation and apoptosis, respectively. From the biological point of view, when fibroblasts enter the wound region, they can differentiate into myofibroblasts, which pull the extracellular matrix even harder and cause the contractions of the wound. Thus, to describe the probability differentiating into myofibroblasts, exponential distribution is still applied with different parameter value $\lambda$, based on the fact that the exponential distribution is memoryless. Similarly, we use the same exponential distribution model to describe the probability of differentiation from a regular fibroblast to myofibroblast with

$$\begin{aligned} \lambda _{\mathrm{myo}}= {\left\{ \begin{array}{ll} 60\times c^2_{\mathrm{TGF}}(\varvec{x},t)+10, &{}\text{ if }\quad c_{\mathrm{TGF}}(\varvec{x},t)>0.01\,\hbox{g}/(\upmu \hbox{m})^3,\\ 0,&{}\text{ otherwise. } \end{array}\right. } \end{aligned}$$

At each time step, we generate a random number $\xi$ from (0, 1). The cell will divide or die or differentiate if and only if $\xi <\mathbb{P}(t\in (t_n,t_n+\Delta t))$ with corresponding $\lambda$.

Additionally, for each cell, it needs some time to grow mature first and then it can divide. We assume that a cell will not divide unless the growth time exceeds some time step threshold, but there is no restriction for cell death.

The related parameter values of cells used in this study are shown in Table 1. The values in the table below are mainly from Chen et al. (2017) and Koppenol (2017). Note that some of the parameter values, such as the dimensions of the cells, are fictitious and that the current paper mainly focusses on evaluating the sensitivity of the model.

**Table 1:** Parameter values of cells which will be used in the calculation of this report

Parameter	Description	Value	Dimension	Reference
$x_0$	Length of the computational domain in *x* direction	120	$\upmu \hbox{m}$	Estimated in this study
$y_0$	Length of the computational domain in *y* direction	80	$\upmu \hbox{m}$	Estimated in this study
$x_w$	Length of the wound region in *x* direction	40	$\upmu \hbox{m}$	Estimated in this study
$y_w$	Length of the wound region in *y* direction	30	$\upmu \hbox{m}$	Estimated in this study
$\Delta t$	Time step	0.1	h	Estimated in this study
$E_s$	Substrate elasticity	50	$\hbox{kg}/(\upmu \hbox{m}\,\hbox{h}^2)$	Dudaie et al. (2015)
$E_c$	Cell elasticity	5	$\hbox{kg}/(\upmu \hbox{m}\,\hbox{h}^2)$	Dudaie et al. (2015)
$F_i$	Traction force between cells	10	$\hbox{kg}\,\upmu \hbox{m}/\hbox{h}^2$	Chen et al. (2017)
*R*	Cell radius	2.5	$\upmu \hbox{m}$	Dudaie et al. (2015)
$\mu$	Cell friction coefficient	0.2	–	Vermolen and Gefen (2015)
$\beta _i$	Mobility of the cell surface	1	$\hbox{h}^{-1}$	Vermolen and Gefen (2015)
$\nu$	Poisson’s ratio of the cell	0.48	–	Estimated in this study
$P_f$	Magnitude of temporary force of regular fibroblasts	8.32	$\hbox{kg}\,\upmu \hbox{m}/\hbox{h}^2$	Koppenol (2017)
$P_m$	Magnitude of temporary force of myofibroblasts	33.28	$\hbox{kg}\,\upmu \hbox{m}/\hbox{h}^2$	Estimated in this study
$Q_0$	Magnitude of plastic force of myofibroblasts	33	$\hbox{kg}\,\upmu \hbox{m}/\hbox{h}^2$	Koppenol (2017)
$\alpha _\rho$	Coefficient related to collagen and fibrin	$10^4$	–	Koppenol (2017)
*v*	Speed of biased movement of cells	2.5	$\upmu \hbox{m}/\hbox{h}$	Koppenol (2017)
$c^0_{\mathrm{PDGF}}$	Initial concentration of PDGF	1	$\hbox{g}/(\upmu \hbox{m})^3$	Koppenol (2017)
$c^0_{\mathrm{tPA}}$	Initial concentration of tPA	1	$\hbox{g}/(\upmu \hbox{m})^3$	Koppenol (2017)
$D_{\mathrm{TGF}}^{\mathrm{min}}$	Minimum diffusion rate of TGF-beta	10.6	$\upmu \hbox{m}^2/\hbox{h}$	Koppenol (2017)
$D_{\mathrm{TGF}}^{\mathrm{max}}$	Maximum diffusion rate of TGF-beta	100.6	$\upmu \hbox{m}^2/\hbox{h}$	Koppenol (2017)
$k_{\mathrm{TGF}}$	Secretion rate of TGF-beta molecules by macrophages	2.5	$\hbox{kg}/(\upmu \hbox{m}^3h)$	Koppenol (2017)
$D_{\mathrm{PDGF}}$	Diffusion rate of PDGF	10	$\upmu \hbox{m}^2/\hbox{h}$	Koppenol (2017)
$D_{\mathrm{tPA}}^{\mathrm{min}}$	Minimum diffusion rate of tPA	0.711	$\upmu \hbox{m}^2/\hbox{h}$	Koppenol (2017)
$D_{\mathrm{tPA}}^{\mathrm{max}}$	Maximum diffusion rate of tPA	14.1	$\upmu \hbox{m}^2/\hbox{h}$	Koppenol (2017)
$k_{\mathrm{tPA}}$	Secretion rate of tPA by damaged endothelial cells	0.5	$\hbox{kg}/(\upmu \hbox{m}^3\hbox{h})$	Koppenol (2017)
$\kappa$	Parameter in Robin’s boundary condition to solve Eq. 6	100	$1/\upmu \hbox{m}$	Estimated in this study
$\kappa _f$	Parameter in Robin’s boundary condition to solve Eq. 9	3	$1/\upmu \hbox{m}$	Estimated in this study
$\delta _\rho$	Degradation rate of fibrin bundles	0.15	$\upmu \hbox{m}^2/\hbox{h}$	Koppenol (2017)
$\sigma _{rw}$	Weight of random walk	0.01	–	Estimated in this study

### Semi-stochastic cell-based model

#### Concentration of signalling molecules

In the model, the fields of the several growth factors are modelled by reaction–diffusion equations of the form:

6 $$\begin{aligned} \frac{\partial c}{\partial t}+\nabla \cdot [\varvec{v}(t,\varvec{x}(t))\cdot c]-\nabla \cdot (D\nabla c)=F, \end{aligned}$$

where *c* is the concentration, *D* is the diffusion rate, which is either a positive constant or a function of fibre and collagen concentration, and $\varvec{v}(t,\varvec{x}(t))$ is the displacement velocity of the substrate that results from the cellular forces exerted on their surroundings. To derive the corresponding Galerkin’s form, Reynold’s transport theorem (Marsden and Tromba 2003; Leal 2007) is applied to dismiss the term with the displacement velocity $\varvec{v}(t,\varvec{x}(t))$. Define material derivative as

$$\begin{aligned} \frac{\mathrm{D}f(\varvec{x}(t),t)}{\mathrm{D}t}=\frac{\partial f(\varvec{x}(t),t)}{\partial t}+\varvec{v}(t,\varvec{x}(t))\cdot \nabla f(\varvec{x}(t),t) \end{aligned}$$

for any tensor field $f(\varvec{x}(t),t)$, where $\varvec{v}(t, \varvec{x}(t))$ is the velocity of the field.

##### **Theorem 1**

*Let* $\Omega _y$ *be a time-dependent domain in* ${\mathbb{R}}^d$, *and let* $\partial \Omega _t$ *be the boundary of* $\Omega _y$, *further, let* *f*, $\frac{\partial f}{\partial t} \in L^2(\Omega _t)$ *be a given function and let* $\mathbf{v}$ *represent the velocity of moving boundary* $\partial \Omega _t$, *then*

$$\begin{aligned} \left. \begin{aligned} \frac{\mathrm{d}}{\mathrm{d}t}\int _{\Omega _t}f(\varvec{x}(t),t)\mathrm{d}\Omega _t=\int _{\Omega _t}\frac{\partial f}{\partial t}(\varvec{x}(t),t)\mathrm{d}\Omega _t\\ +\int _{\partial \Omega _t}f(\varvec{x}(t),t)\varvec{v}(t,\varvec{x}(t))\cdot \varvec{n}\mathrm{d}\Gamma , \end{aligned} \right. \end{aligned}$$

*where* $\varvec{n}$ *is the outward-pointing unit normal vector*.

Robin’s boundary conditions are used here since it models a balance between the diffusive flux from the domain of computation into and the mass transfer into the surroundings around the domain of computation. The symbol $\kappa$, which is non-negative, represents the mass transfer coefficient. Note that as $\kappa \rightarrow 0$ then the Robin condition tends to a homogeneous Neumann condition, which represents no flux (hence isolation). Whereas as $\kappa \rightarrow \infty$ represents the case that $c \rightarrow 0$ on the boundary, which, physically, is reminiscent to having an infinite mass flow rate at the boundary into the surroundings. The Robin condition, also referred to as a mixing boundary condition, is able to deal with both these two limits and all cases between these limits.

With Robin’s boundary condition and applying Reynold’s theorem(Theorem  1), the Galerkin’s form of Eq. 6 is

$$\begin{aligned} \left\{ \begin{aligned}&\text {Find } c_h \in C^1_h((0,T]\times H_h^1(\Omega _t)) \cap C_h^0([0,T]\times H_h^1(\Omega _t)),\\&\text {such that}\\&\frac{\mathrm{d}}{\mathrm{d}t}\int _{\Omega _t}c_h\phi _h \mathrm{d}\Omega _t+\int _{\Omega _t}D\nabla \phi _h\nabla c_hd\Omega _t+\int _{\partial \Omega _t}\kappa c_h\phi _hd\Gamma _t\\&=\int _{\Omega _t}F\phi _h \mathrm{d}\Omega _t,\\&\forall \phi _h\in C_h^1((0,T]\times H_h^1(\Omega _t)) \cap C_h^0([0,T]\times H_h^1(\Omega _t)), \end{aligned} \right. \end{aligned}$$

where $c_h(t)$ is the numerical solution of the concentration at time *t*.

Furthermore, linear triangular basis functions are utilized to derive Galerkin’s form. Since the basis function is attached to vertices, it can be concluded that for the basis function at any mesh point *i*, we have $\frac{\mathrm{D}\phi _j(\varvec{x}(t))}{\mathrm{D}t}=0$ (Dziuk and Elliott 2007) for all $j\in \{1,\ldots N_h\}$ where $N_h$ is the number of nodal points in the domain. In our computational implementation, the backward Euler method is applied to integrate over time, i.e. the Galerkin’s form above is altered into

$$\begin{aligned} \left\{ \begin{aligned}&\text{ Find }\,c_h \in C^1((0,T]\times H_h^1(\Omega _t)) \cap C^0([0,T]\times H_h^1(\Omega _t)),\\&\text{ such } \text{ that }\\&\frac{1}{\Delta t} \left( \int _{\Omega _{t+\Delta t}}c_h(t+\Delta t)\phi _h(t+\Delta t) d\Omega _{t+\Delta t}\right. \\&\left. -\int _{\Omega _t}c_h(t)\phi _h(t) \mathrm{d}\Omega _t\right) \\&+\int _{\Omega _{t+\Delta t}}D\nabla \phi _h(t+\Delta t)\nabla c_h(t+\Delta t)\mathrm{d}\Omega _{t+\Delta t}\\&+\int _{\partial \Omega _{t+\Delta t}}\kappa c_h(t+\Delta t)\phi _h(t+\Delta t)\mathrm{d}\Gamma _{t+\Delta t}\\&=\int _{\Omega _{t+\Delta t}}F\phi _h(t+\Delta t) \mathrm{d}\Omega _{t+\Delta t},\\&\forall \phi _h\in C^1((0,T]\times H_h^1(\Omega _t)) \cap C^0([0,T]\times H_h^1(\Omega _t)), \end{aligned} \right. \end{aligned}$$

where $c_h(t)$ is the numerical solution of the concentration at time *t*. To distinguish between the cytokines, we add the subscript “PDGF”, “TGF” or “tPA” if it is necessary. As for the velocity of the environment (i.e. the extracellular matrix) where all the biological elements live on, we, indeed, compute the displacement from the solution of the balance of momentum. Subsequently, the velocity is post-processed from the numerical time derivative, where following Madzvamuse and George (2013) $\mathbf{v}(t,\mathbf{x}(t)) = \frac{D \mathbf{u(t,\mathbf{x}(t))}}{Dt}$, and hence we get

$$\begin{aligned} \varvec{v}(t,\varvec{x}(t)) \approx \frac{\mathbf{u}(t+\Delta t,\mathbf{x}(t+\Delta t)) - \mathbf{u}(t,\varvec{x}(t))}{\Delta t}. \end{aligned}$$

The platelet-derived signalling molecules have been regenerated by the platelets. We assume that the platelets are no longer active and therefore the platelet-derived signalling molecules are only subject to diffusion. Alternative processes such as regeneration and decay are neglected. Thus, the equation to determine the concentration of PDGF is given by

7 $$\begin{aligned} \frac{\partial c_{\mathrm{PDGF}}}{\partial t}+\nabla \cdot [c_{\mathrm{PDGF}}\cdot \varvec{v}(t,\varvec{x}(t))]-D_{\mathrm{PDGF}}\Delta c_{\mathrm{PDGF}}=0, \end{aligned}$$

where $D_{\mathrm{PDGF}}$ is the diffusion rate given in Table 1.

Regarding the concentration of TGF-beta, it is widely documented that macrophages are the one of the main sources (Boon et al. 2016; Cumming et al. 2009; Enoch and Leaper 2008). Therefore, each viable macrophage is a point source of TGF-beta and hence Dirac delta distributions are used. Furthermore, we assume that the diffusion rate of the signalling molecule is linearly dependent on the local density of the fibrin molecules (Deuel et al. 1982). All these assumptions yield the following equation to determine the concentration of TGF-beta:

8 $$\begin{aligned} \begin{aligned}&\frac{\partial c_{\mathrm{TGF}}}{\partial t}+\nabla \cdot [c_{\mathrm{TGF}}\cdot \varvec{v}(t, \varvec{x}(t))]\\&-\nabla \cdot [D_{\mathrm{TGF}}(\rho ^f)\nabla c_{\mathrm{TGF}}]=k_{\mathrm{TGF}}\sum _{i=1}^{T_M(t)}\delta (\varvec{x}-\varvec{x_i}), \end{aligned} \end{aligned}$$

with

$$\begin{aligned} D_{\mathrm{TGF}}(\rho ^f)=(\alpha _\rho \rho ^f)D_{\mathrm{TGF}}^{\mathrm{min}}+(1-\alpha _\rho \rho ^f)D^{\mathrm{max}}_{\mathrm{TGF}}. \end{aligned}$$

Here, $D_{\mathrm{TGF}}^{\min }$ and $D_{\mathrm{TGF}}^{\max }$ are the minimum and maximum diffusion rate of tPA, $k_{\mathrm{TGF}}$ is the secretion rate of TGF-beta of each macrophage and $\alpha _\rho$ is a given positive constant. All the parameter values are given in Table 1.

#### Force balance

The (myo)fibroblasts exert pulling forces on their immediate surroundings in the extracellular matrix. These forces are directed towards the cell centre and cause local displacements and deformation of the extracellular matrix. The combination of all these forces causes a net contraction of the tissue around the region where the fibroblasts are actively exerting forces. The (myo)fibroblast exert pulling forces on their immediate environment. Next to these forces, they change the local environment in a way that residual forces remain after their presence. Therefore, we consider two types of forces: temporary force ($\varvec{f}_t$) and plastic force ($\varvec{f}_p$).

As it is stated before, the force results in the deformation of the skin surrounding the wound. Neglecting inertia, the balance of momentum in $\Omega _t$ reads as:

9 $$\begin{aligned} -\nabla \cdot \underline{\underline{\sigma }}=\varvec{f}_t+\varvec{f}_p. \end{aligned}$$

From a mechanical point of view, we treat the computational domain as a continuous linear isotropic domain. Further, as a result of the presence of liquid phases in the tissue, the mechanical balance is also subject to viscous, that is friction, effects. Therefore, we use Kelvin–Voigt’s viscoelastic dashpot model, which in essence reads as

$$\begin{aligned} \underline{\underline{\sigma }}=\underline{\underline{E}}\underline{\underline{\epsilon }}+\underline{\underline{\mu }}\dot{\underline{\underline{\epsilon }}}, \end{aligned}$$

where $\underline{\underline{E}}$ and $\underline{\underline{\mu }}$ are linear tensors and they will be specified now. We decompose the total stress by

10 $$\begin{aligned} \underline{\underline{\sigma }}=\underline{\underline{\sigma }}_{\mathrm{elas}}+\eta \underline{\underline{\sigma }}_{\mathrm{visco}} \end{aligned}$$

where $\eta$ is the weight of the viscoelasticity stress tensor, $\underline{\underline{\sigma }}_{\mathrm{elas}}$ is

11 $$\begin{aligned} \underline{\underline{\sigma }}_{\mathrm{elas}}=\frac{E}{1+\nu }\left\{ \underline{\underline{\epsilon }}+tr(\underline{\underline{\epsilon }})\left[ \frac{\nu }{1-2\nu }\right] \underline{\underline{I}}\right\} , \end{aligned}$$

and $$\underline{\underline{\sigma }}_{\mathrm{visco}}$$ is

12 $$\begin{aligned} \underline{\underline{\sigma }}_{\mathrm{visco}}=\frac{E}{2(1+\nu )}\dot{\underline{\underline{\epsilon }}}+\frac{2}{3}\times \frac{E}{2(1+\nu )}\nabla \cdot \dot{\varvec{u}}\underline{\underline{I}}. \end{aligned}$$

In the above equations, *E* is Young’s modulus of the domain, $\nu$ is Poisson’s ratio and $\underline{\underline{\epsilon }}$ is the infinitesimal strain tensor, that is,

13 $$\begin{aligned} \underline{\underline{\epsilon }}=\frac{1}{2}\left[ \nabla \varvec{u}+(\nabla \varvec{u})^T\right] . \end{aligned}$$

The above PDE provides a good approximation if the strains are relatively small. Combined with Robin’s boundary condition

$$\begin{aligned} \underline{\underline{\sigma }}\cdot \varvec{n}+\kappa _f\varvec{u}=\varvec{0}, \end{aligned}$$

where $\kappa _f$ is a non-negative constant representing a spring force constant between the computational domain and its far away surroundings, the displacement $\varvec{u}$ of the extracellular matrix where all the biological components are, can be determined by Eq. 9. Note that if $\kappa _f\rightarrow \infty$, then $\varvec{u}\rightarrow \varvec{0}$ which represents a fixed boundary, and $\kappa _f\rightarrow 0$ represents a free boundary in the sense that no external force is exerted on the boundary.

**Temporary Force** Temporary forces are characterized by (myo)fibroblasts exerting forces from the boundary of the cell onto their immediate surroundings. Once there is no cell, the deformation caused by the forces will disappear. For cell *i*, the cell boundary $\Gamma ^i$ is divided into finite line segments in two-dimensional case. We assume that an inward directed force is exerted as a point force at the midpoint of every line segment in the normal direction to the line segment. The total force is a linear combination of every force at every segment. Hence, at time *t*, the total temporary force is expressed by

14 $$\begin{aligned} \varvec{f}_t(t)=\sum _{i=1}^{T_N(t)}\sum _{j=1}^{N_S^i} P(\varvec{x},t)\varvec{n}(\varvec{x}) \delta (\varvec{x}-\varvec{x}_j^i(t))\Delta \Gamma _N^{i,j}, \end{aligned}$$

where $T_N(t)$ is the number of cells at time *t*, $N_S^i$ is the number of line segments of cell *i*, $P(\varvec{x},t)$ is the magnitude of the pulling force exerted at point $\varvec{x}$ and time *t* per length, $\varvec{n}(\varvec{x})$ is the unit inward pointing normal vector (towards the cell centre) at position $\varvec{x}$, $\varvec{x}_j^i(t)$ is the midpoint on line segment *j* of cell *i* at time *t* and $\Delta \Gamma _N^{i,j}$ is the length of line segment *j*. This method is also used in fluid dynamics and is known as the immerse boundary method. Theoretically, as $N_S^i\rightarrow \infty$, i.e. $\Delta \Gamma _N^{i,j}\rightarrow 0$, Eq. 14 becomes (Boon et al. 2016)

15 $$\begin{aligned} \varvec{f}_t(t)=\sum _{i=1}^{T_N(t)}\int _{\partial \Omega _N^i} P(\varvec{x},t)\varvec{n}(\varvec{x}) \delta (\varvec{x}-\varvec{x}^i(t))\mathrm{d}\Gamma _N^{i}, \end{aligned}$$

Here, $\varvec{x}^i(t)$ is a point on the cell boundary of cell *i* at time *t*.

For the sake of computational efficiency, each cell is divided into three boundary segments (Peng and Vermolen 2019). Therefore, $N_S^i=3$ in Eq. 14.

**Plastic Force**

Plastic forces occur as a result of the changes that myofibroblasts inflict on their immediate surroundings. On the one hand, these forces are caused by large deformations, but also by the chemical decomposition of collagen chains into shorter chains. The shortening of the chains causes stresses and strains in the cross-linked network of collagen molecules. The modelling of the permanent forces proceeds similarly to the modelling of the temporary forces with the only difference that the forces are not acting on the cellular boundary but on the boundary of an element of the finite-element mesh. Similarly to the temporary forces, the total plastic force on the boundary segments of the elements of the mesh at time *t* is expressed by Boon et al. (2016)

16 $$\begin{aligned} \varvec{f}_p=\sum _{i=1}^{N_E}\sum _{e=1}^{N_e^i}Q(\tau ^i) \varvec{n}(\varvec{x})\delta (\varvec{x}-\varvec{x}_e^i(t)) \Delta \Gamma _E^{i,e}, \end{aligned}$$

where $N_E$ is the total number of mesh triangular elements, $N_e^i$ is the total number of edges of the mesh triangular elements, $Q(\tau ^i)$ is the magnitude of the plastic force density at efficient exposure time $\tau ^i$ of myofibroblast, $\varvec{n}(\varvec{x})$ is the unit inward pointing normal vector, $\varvec{x}_e^i(t)$ is the midpoint and $\Delta \Gamma _E^{i,e}$ is the length of the edge, respectively. We note that this framework is sufficiently general to extend to different element shapes.

Note that here the magnitude of the force is a function of the exposure time $\tau ^i$ of the element by the myofibroblasts, denoted by $Q(\tau ^i)$. In Koppenol (2017), it was hypothesized that the effective exposure of each element on the finite-element mesh was expressed by

17 $$\begin{aligned} \frac{\mathrm{d}\tau ^i}{\mathrm{d}t}=c_{\mathrm{TGF}}(\varvec{x}_E^i) (1-\alpha _\rho \rho ^c(\varvec{x}_E^i)) \frac{A(\Omega _C\cap \Omega _{e_i})}{A(\Omega _{e_i})}, \end{aligned}$$

where $\varvec{x}_E^i$ is the central point of the mesh element *i*, $\rho ^c(\varvec{x}_E^i)$ is the density of collagen at $\varvec{x}_E^i$, $A(\Omega _C\cap \Omega {e_i})$ is the area of the intersection of the cell and mesh element *i* and $A(\Omega _{e_i})$ is the area of mesh element *i*. Note that the area ratio $\frac{A(\Omega _C\cap \Omega _{e_i})}{A(\Omega _{e_i})}$ will not exceed unity. However, to implement into the computation, it is hard to calculate this ratio explicitly. Therefore, we rewrite Eq. 17. Since it is not straightforward from a computational point of view to compute the area of overlap, we simplify this operation by

18 $$\begin{aligned} \frac{\mathrm{d}\tau ^i}{\mathrm{d}t}=c_{\mathrm{TGF}}(\varvec{x}_E^i) (1-\alpha _\rho \rho ^c(\varvec{x}_E^i))N_{\mathrm{myo}}^i, \end{aligned}$$

where $N_{\mathrm{myo}}^i$ is the number of myofibroblasts that the central point of mesh element *i* is in.

The magnitude of the plastic force density is a function of exposure time $\tau ^i$:

19 $$\begin{aligned} Q(\tau ^i)=Q_{\mathrm{max}}(1-e^{-\tau ^i}), \end{aligned}$$

where $Q_{\mathrm{max}}$ is the maximal magnitude of the plastic force density.

#### Orientation of tissues

Dallon et al. (1999) and Cumming et al. (2009) introduced vector-based representations of collagen and fibrin. The approach by Cumming et al. (2009) is tensor based, and since this makes it straightforward to incorporate the impact from the orientation on the migration path of the cells, we use the formalism by Cumming et al. (2009).

The orientation of the bundles and the density of collagen molecules at position $\varvec{x}$ and time *t* are determined by the general symmetric tensor:

20 $$\begin{aligned} \underline{\underline{\Omega }}^k(\varvec{x},t)=\int _{0}^{\pi }\varvec{p}(\theta ) \varvec{p}(\theta )^T\rho (\varvec{x},t,\theta )d\theta , \end{aligned}$$

where *k* indicates the type of the tissue: *f* for fibrin and *c* for collagen, $\varvec{p}(\theta )^T=[\cos \theta ,\sin \theta ]$ is the unit vector in the direction $\theta$, and $\rho (\varvec{x},t,\theta )$ is the density of collagen at position $\varvec{x}$ and time *t*. Note that in this manuscript, $\underline{\underline{\Omega }}^k$ and $\Omega ^{k}_{i,j}$ denotes the tensor of fibrin or collagen and the entries in the tensor, respectively. The density of collagen can be determined by the trace of $\underline{\underline{\Omega }}^k$, that is, the summation of the eigenvalues or diagonal entries. Since the tensor is symmetric positive definite, it can be decomposed as the sum of weighed outer products of orthonormal eigenvectors, which in the two-dimensional case gives:

$$\begin{aligned} \underline{\underline{\Omega }}^k(\varvec{x},t)&=\lambda _1(\varvec{x},t)\varvec{v_1}(\varvec{x},t)\varvec{v_1}(\varvec{x},t)^T\\&\quad +\lambda _2(\varvec{x},t)\varvec{v_2}(\varvec{x},t)\varvec{v_2}(\varvec{x},t)^T, \end{aligned}$$

where $\lambda _1(\varvec{x},t)$ and $\lambda _2(\varvec{x},t)$ are eigenvalues. The orientation of the eigenvectors illustrates the direction of alignment, and

$$\begin{aligned} e=1-\frac{\lambda _{\mathrm{min}}}{\lambda _{\mathrm{max}}}, \end{aligned}$$

represents the degree of anisotropy. Note that if both eigenvalues are equal, then the dermal layer is completely isotropic, which renders $e = 0$.

We incorporate the breakdown of the provisional fibrin-based extracellular matrix into the model. Assuming the degradation rate of the fibrin bundles to be determined by the concentration of tPA and the concentration of the fibrin bundles themselves (Koppenol 2017), then for any entry in the fibrin orientation tensor, we have

21 $$\begin{aligned} \frac{\partial \Omega ^f_{ij}}{\partial t}+\nabla \cdot [\Omega ^f_{ij}\cdot \varvec{v}(t,\varvec{x}(t))] =-\delta _\rho [c_{\mathrm{tPA}}\Omega ^f_{ij}], \end{aligned}$$

for any $i,j\in \{1,2\}$ and $\delta _\rho$ represents the degradation rate of fibrin bundles. Note that the $\varvec{v}$-term accounts for the mesh movement velocity.

The concentration of tPA is assumed that it is only secreted on the edge between the injured and uninjured regions. In addition, based on Koppenol (2017), the diffusion rate of tPA is related to the local concentration of fibrin molecules. Hence, the equation below is used to derive the concentration of tPA:

22 $$\begin{aligned} \begin{aligned}\frac{\partial c_{\mathrm{tPA}}}{\partial t}+\nabla \cdot [c_{\mathrm{tPA}}\cdot \varvec{v}(t,\varvec{x}(t))]&=\nabla \cdot [D_{\mathrm{tPA}}(\rho ^f)\nabla c_{\mathrm{tPA}}]\\&\quad +k_{\mathrm{tPA}}\delta _{\partial \Omega _{w}}, \end{aligned} \end{aligned}$$

where

$$\begin{aligned} D_{\mathrm{tPA}}(\rho ^f)=(\alpha _\rho \rho ^f)D_{\mathrm{tPA}}^{\min } +(1-\alpha _\rho \rho ^f)D_{\mathrm{tPA}}^{\max }. \end{aligned}$$

Here, $D_{\mathrm{tPA}}^{\min }$ and $D_{\mathrm{tPA}}^{\max }$ are the minimum and maximum diffusion rate of tPA, $k_{\mathrm{tPA}}$ is the secretion rate of tPA by damaged endothelial cells and $\alpha _\rho$ is a given positive constant. We define the distributions $\delta _{\partial \Omega _{w}}$ as

$$\begin{aligned} \delta _{\partial \Omega _{w}}(\varvec{x})\left\{ \begin{aligned}&=0,\quad \text{ if }\quad \varvec{x}\notin \partial \Omega _{w},\\&>0,\quad \text{ if }\quad \varvec{x}\in \partial \Omega _{w}, \end{aligned}\right. \end{aligned}$$

such that for any region $A\subset \Omega$, there is

$$\begin{aligned} \int _A\delta _{\partial \Omega _{w}}(\varvec{x})\mathrm{d}\Omega =\frac{\mu (A\cap \partial \Omega _{w})}{\mu (\partial \Omega _{w})}, \end{aligned}$$

in which the measure $\mu :\partial \Omega _{w}\rightarrow \mathbb{R}^+$ is the length of the curve $\partial \Omega _{w}$.

The collagen bundles are deposited by the (myo)fibroblasts in the direction of **active** migration. Since the cells are much smaller than the computational domain, we use Dirac Delta functions to model the secretion of the collagen bundles. Furthermore, the secretion rate depends on the amount of total density including fibrin and collagen. This amounts (see also Koppenol 2017; Cumming et al. 2009; Boon et al. 2016)

23 $$\begin{aligned} \left. \begin{aligned}&\frac{\partial \Omega ^c_{ij}}{\partial t}+\nabla \cdot [\Omega ^c_{ij}\cdot \varvec{v}(t,\varvec{x}(t))]=\sum _{k=1}^{T_N(t)}\left( 1-\alpha _\rho [\rho ^f(\varvec{x}_N^k(t))\right. \\&\left. \quad +\rho ^c(\varvec{x}_N^k(t))]\right) [\varvec{r}_N^k(t)(\varvec{r}_N^k(t))^T]_{ij}\delta (\varvec{x}-\varvec{x}_N^k(t)), \end{aligned} \right. \end{aligned}$$

where $\varvec{r}_N^k(t)=(\mathrm{d}\varvec{x}^k_N(t)-\varvec{v}\mathrm{d}t)/\Vert \mathrm{d}\varvec{x}^k_N(t)-\varvec{v}\mathrm{d}t\Vert$ is the unit vector of the direction of active displacement of (myo)fibroblast *k* at time *t* for any $i,j\in \{1,2\}$, and $\varvec{x}_N^k(t)$ is the centre position of (myo)fibroblast with index *k*, see Eq. (25).

#### Displacement of various cell phenotypes

Generally speaking, to determine the position of any cell in the semi-stochastic cell-based model is a combination of interactions between cells, namely repulsion when there is mechanic contact for any two cells; chemotaxis, which is related to the signalling molecules; passive convection due to the displacement of the substrate and random walk. In addition, random walk, is also embedded within the displacement model. Here, $d \mathbf{W}(t)$ represents a vector Wiener process in which the contributions to the different coordinate directions are treated as independent events. This Wiener process models random walk of the cell. Note that $\sigma _{rw} = \sqrt{2 D_c}$, where $D_c$ represents the diffusion coefficient of the cell phenotype of cell *i*.

For the immune cells, chemotaxis is associated with PDGF, then the displacement of the centre position of *i*-th macrophage is given by

24 $$\begin{aligned} \left. \begin{aligned} \mathrm{d}\varvec{r}_i(t)&=\alpha _i\hat{M}(\varvec{r_i})\varvec{\hat{z}_i}\mathrm{d}t+\mu _{c}\frac{\nabla c_{\mathrm{PDGF}}}{\Vert \nabla c_{\mathrm{PDGF}}\Vert +\gamma }\mathrm{d}t+\varvec{v}\mathrm{d}t\\&\quad +\sigma _{rw} \mathrm{d}\varvec{W}(t),\quad \text{ for } \text{ all }~i\in \left\{ 1,\ldots ,n\right\} , \end{aligned}\right. \end{aligned}$$

where $\mu _{c}$ is the constant representing the weight of chemotaxis, which is expressed by

$$\begin{aligned} v\left( 1-\alpha _\rho \frac{\rho ^f+\rho ^c}{2}\right) , \end{aligned}$$

here, *v* is the speed of biased movement of cells, $c_{\mathrm{PDGF}}$ is the concentration of PDGF which is initially high in injured region and low in uninjured region, $\gamma$ is a small positive constant to prevent the denominator being zero and $\varvec{v}$ is the displacement velocity of the substrate, which follows from solving the momentum balance.

For (myo)fibroblasts, except the concentration part is related to TGF-beta, the rest is the same as for macrophages, i.e. for *i*-th (myo)fibroblast, the new position is derived by

25 $$\begin{aligned} \left. \begin{aligned} \mathrm{d}\varvec{r}_i(t)&=\alpha _i\hat{M}(\varvec{r_i})\varvec{\hat{z}_i}dt+\mu _{c}\left\{ \left[ 1-\alpha _\rho \rho ^c(\varvec{r}_i(t))\right] \underline{\underline{I}}\right. \\&+\left. \left[ \alpha _\rho \rho ^c(\varvec{r}_i(t))\underline{\underline{\Omega }}^c(\varvec{r}_i(t))\right] \right\} \frac{\nabla c_{\mathrm{TGF}}}{\Vert \nabla c_{\mathrm{TGF}}\Vert +\gamma }\mathrm{d}t\\&+\varvec{v}\mathrm{d}t+\sigma _{rw} \mathrm{d}\varvec{W}(t),\quad \text{ for } \text{ all }\,i\in \left\{ 1,\ldots ,n\right\} , \end{aligned} \right. \end{aligned}$$

where $\mu _{c}$ is the same expression as before, $c_{\mathrm{TGF}}$ is the concentration of TGF-beta secreted by macrophages.

In Eqs. (24) and (25), $\alpha _i$ stands for a cell motility parameter with dimension $\left[ \frac{h\cdot \mu m}{kg}\right]$ of which the expression is

$$\begin{aligned} \alpha _i=\frac{\beta _i R^3}{\mu F_i}, \end{aligned}$$

as outlined in Gefen (2010). Here, $\beta _i$ represents a coefficient for the mobility of the portion of the cell surface that is in physical contact with the substrate of another cell, and dimensionless parameter $\mu$ is the friction coefficient between the cell surface and the underlying substrate (Gefen 2010). The values of the parameters are listed in Table 1.

### Initial settings of the model

Initially, there are no myofibroblasts. The TGF-beta concentration determines the differentiation rate of fibroblasts to myofibroblasts. Macrophages are uniformly distributed on the edge between wound and the undamaged region. The fibroblasts are initially distributed randomly in the undamaged region.

It is assumed that the concentration of PDGF is higher in the injured region and lower in the undamaged region. In order to specify the concentration, we introduce the following indicator function

26 $$\begin{aligned} {\mathbb{I}}_{\Omega _w}= {\left\{ \begin{array}{ll} 1,&{}\varvec{x}\in \Omega _{w},\\ 0,&{}\varvec{x}\notin \Omega _{w}, \end{array}\right. } \end{aligned}$$

where $\Omega _{w}$ is the wound region as a subdomain in the computational domain. Subsequently, the initial setting of PDGF is

$$\begin{aligned} c^0_{\mathrm{PDGF}}(\varvec{x})={\mathbb{I}}_{\Omega _w}c^0_{\mathrm{PDGF}}, \end{aligned}$$

where $c^0_{\mathrm{PDGF}}$ is given in Table 1. Since TGF-beta is mainly secreted by macrophages, the initial condition of it is zero everywhere over the computational domain, that is,

$$\begin{aligned} c^0_{\mathrm{TGF}}(\varvec{x})=0, \quad \hbox{in}\quad \Omega _t. \end{aligned}$$

As for tPA, the concentration is higher on the edge between injured and uninjured region and lower in the rest part:

$$\begin{aligned} c^0_{\mathrm{tPA}}(\varvec{x})= {\left\{ \begin{array}{ll} c^{\Gamma _w}_{\mathrm{tPA}}, &{} \varvec{x}\in \partial \Omega _{w},\\ 0,&{}\text {otherwise,} \end{array}\right. } \end{aligned}$$

where $\partial \Omega _{w}$ is the edge between wound and healthy skin, and $c^{\Gamma _w}_{\mathrm{tPA}}$ is given in Table 1.

Initially, the material is assumed to be in mechanical equilibrium. Therefore, there are no stresses and strains.

It is assumed that the initial collagen and fibrin are isotropic. Therefore, we have

27 $$\begin{aligned} \underline{\underline{\Omega }}^f_0=\frac{{\mathbb{I}}_{\Omega _w}}{2\alpha _\rho }\underline{\underline{I}}=\frac{{\mathbb{I}}_{\Omega _w}}{2\alpha _\rho } \begin{bmatrix} 1&{}0\\ 0&{}1 \end{bmatrix}, \end{aligned}$$

and

28 $$\begin{aligned} \underline{\underline{\Omega }}^c_0=\frac{1-{\mathbb{I}}_{\Omega _w}}{2\alpha _\rho }\underline{\underline{I}}=\frac{1-{\mathbb{I}}_{\Omega _w}}{2\alpha _\rho } \begin{bmatrix} 1&{}0\\ 0&{}1 \end{bmatrix}, \end{aligned}$$

where $\mathbb{I}_{\Omega _w}$ is the indicator function that was introduced in Eq. (26) and $\alpha _\rho$ is a positive constant.

## Numerical results

**Fig. 3:**
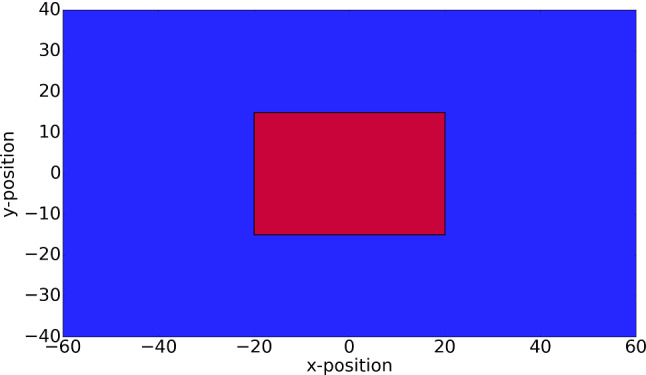
The computational domain in two dimensions is $(-60,60)\times (-40,40)$, in which the wound region is $(-20, 20)\times (-15,15)$ depicted by red and the undamaged tissue is depicted by blue

The finite-element method has been embedded within the FEniCS (Langtangen and Logg 2016) package that has been implemented in Python. Based on the size of the computational domain (see Fig. 3), averagely each time iteration takes 5–7 s depending on the total number of cells in the domain as well. Since the computational efficiency is not the focus of this manuscript, the algorithm has not been optimized yet. Computations have been done on an Intel(R) Core(TM) i7-6500U CPU @ 2.50 GHz computer.

### Displacements of cells

**Fig. 4:**
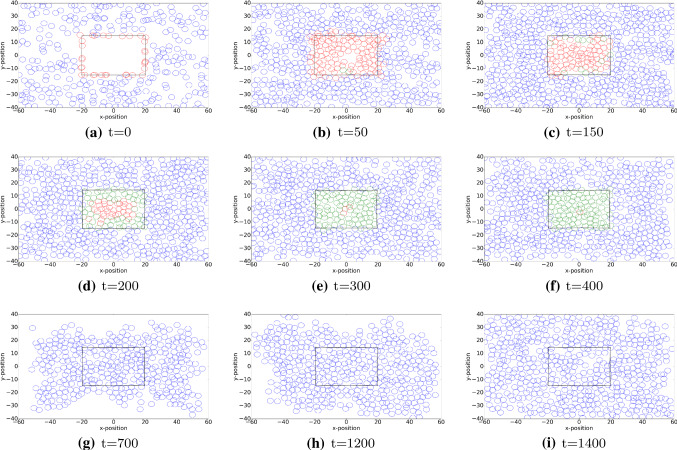
The plots show the positions of three categories of cells in the computational domain. Red, blue and green circles represent macrophages, regular fibroblasts and myofibroblasts, respectively. The rectangular shape in the middle is the wound region at different time. The parameter values used to solve the partial differential equations are taken from Table 1

Firstly, we study the dynamics of the different cell phenotypes as a function of time. In Fig. 4, we show the different cell phenotypes that are indicated by different colours. The red, green and blue circles represent the immune cells, myofibroblasts and fibroblasts, respectively. In the early stages, it can be seen that the fibroblasts are only distributed over the undamaged domain and that the immune cells are scattered over the interface between the wound and undamaged area. As time proceeds, the immune cells migrate into the wound where they release the growth factor TGF-beta. The build-up of the TGF-beta triggers the ingress of fibroblasts, which in turn differentiate to myofibroblasts. In the intermediate stages, it can be seen that the wound region contains myofibroblasts, which are responsible for the largest portion of temporary forces and displacements and which also facilitate the permanent displacements. During this stage, the immune cells are subject to apoptosis and therewith disappear. At the later stages, the myofibroblasts are subject to apoptosis and the wound region is occupied by fibroblasts.

**Fig. 5:**
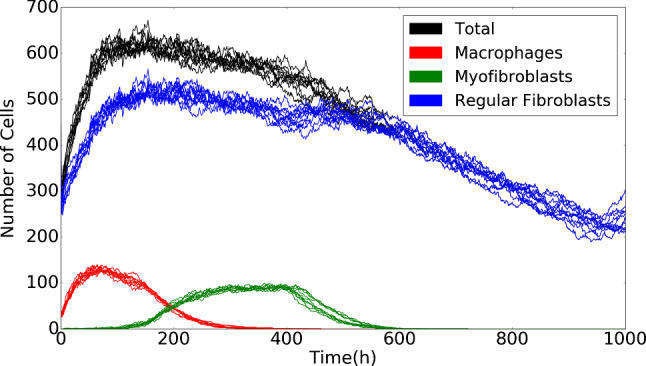
The plot shows the number of various phenotypes of cells changes over time with multiple simulations. The blue, red and green curves represent regular fibroblasts, macrophages and regular myofibroblasts, respectively. The black curves represent the total count of all the cells in the computational domain. The parameter values used to solve the partial differential equations are taken from Table 1

In Fig. 5, the cell counts for the various phenotypes are shown. The figure shows 10 runs of simulations. It can be seen that in the early stages the immune cells accumulate and the damaged region is cleaned. The immune cells trigger the ingress of fibroblasts, and upon decreasing cell counts of immune cells, the counts of myofibroblasts accumulate. Subsequently, after having regenerated collagen, the counts of myofibroblasts decrease as a result of apoptosis. The increased counts of fibroblasts drop back to numbers that are reminiscent to the undamaged state.

### Concentration of signalling molecules and density of tissue bundles

**Fig. 6:**
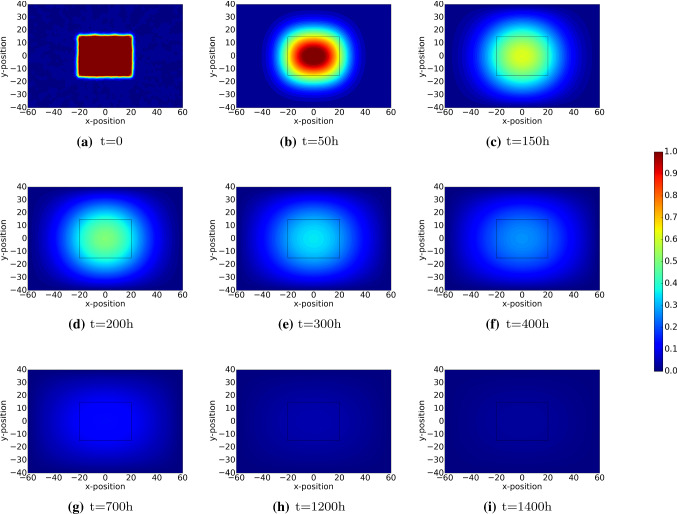
The plots show the dynamics of PDGF, which induces the displacements of macrophages. The results are derived by solving Eq. 7 with Robin’s boundary condition. The parameter values used to solve the partial differential equations are taken from Table 1

Initially, the PDGF concentration is maximal near the interface between the undamaged region and wounded region. This can be seen in Fig. 6a. As time proceeds, diffusion flattens the profiles of the PDGF concentration and the concentration tends to zero due to diffusion to the outer surroundings. The TGF-beta is regenerated by the immune cells and hence as long as the immune cells are in the wounded region, then the concentration increases. At later stages, the immune cells disappear and therewith the concentration of TGF-beta no longer increases and starts decreasing and flattening as a result of diffusion. The gradient of the TGF-beta makes the fibroblasts migrate to the wound site. The peak of the TGF-beta concentration is at around 100 h post wounding, which is in line with the experimental observations from Dallon et al. (2001).

**Fig. 7:**
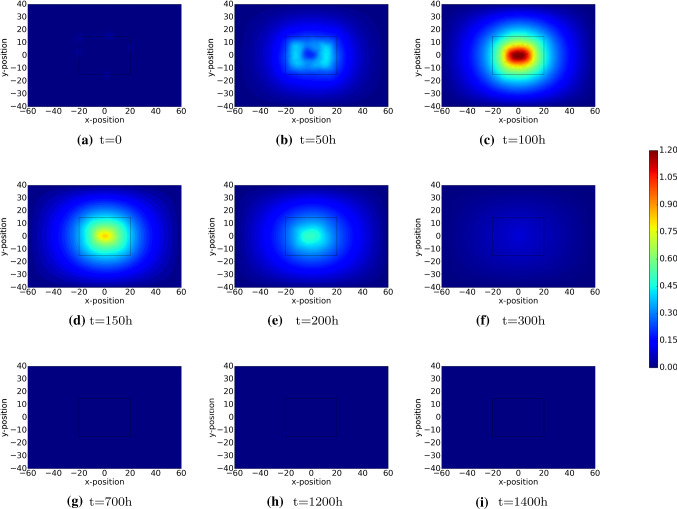
The plots show the dynamics of TGF-$\beta$, which induces the displacements of (myo)fibroblasts. The results are derived by solving Eq. 8 with Robin’s boundary condition. The parameter values used to solve the partial differential equations are taken from Table 1

As cells are proliferating, migrating and subject to apoptosis, they secrete chemicals like PDGF and TGF-beta. Hence, next to diffusion, these chemicals are subject to regeneration and decay. Since PDGF is present initially as a result of platelets, this agent is active during a relatively short time since the platelets are subject to apoptosis shortly after wounding. Due to diffusion into the tissue and due to lack of regeneration, PDGF vanishes shortly after wounding. The immune cells are attracted towards the wound region by PDGF and the immune cells secrete TGF-beta. Hence, the peak in TGF-beta follows the peak of the PDGF. After a while, the immune cells disappear, which also makes the source of TGF-beta vanish and hence due to long distance diffusion, the concentration of TGF-beta decays down to zero. The dynamics of the fields of PDGF and TGF-beta can be seen in Figs. 6 and 7, respectively.

**Fig. 8:**
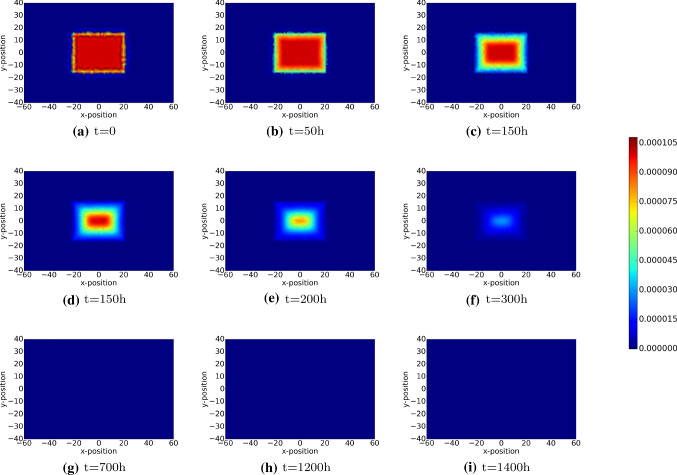
The plots show the density of fibrin bundles over time. The results are derived by solving Eq. 21. The parameter values used to solve the partial differential equations are taken from Table 1

**Fig. 9:**
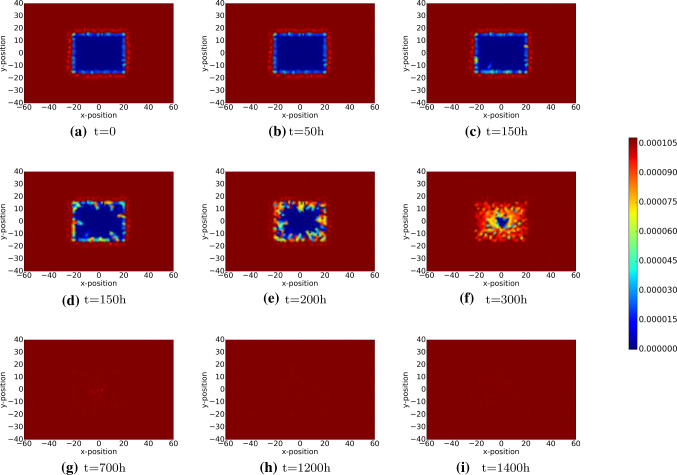
The plots show the density of collagen bundles over time. The results are derived by solving Eq. 23. The parameter values used to solve the partial differential equations are taken from Table 1

The damage on skin is characterized by loss of extracellular matrix and cells. A pivotal aspect of wound healing is the restoration of collagen. Before collagen is deposited, first a fibrin network is established by the platelets by means of polymerization. In the modelling, we assume the fibrin network to have laid down by the platelets. Initially, we assume the fibrin network (blood clot) to exist in the damaged region. In the first stage, the fibrin network is decomposed by the agent tPA. Subsequently, the fibrin network is replaced with collagen by the fibroblasts. The orientation of the deposited collagen is determined by the direction of migration of the (myo)fibroblasts. The dynamics of the fibrin and collagen density are shown in Figs. 8 and 9, respectively.

Figure 8 illustrates the initial occupance of the wound region by the fibrin network and in the undamaged region there is no fibrin. Gradually, the fibrin network is subject to decay as a result of the tPA agent. Figure 9 shows that initially the collagen density is zero in the wounded region, whereas the undamaged region possesses the equilibrium value of collagen. As time proceeds, the fibrin network decays in the wounded region, whereas the collagen density increases due to regeneration by the (myo)fibroblasts. The orientation of the collagen bundles guide the migration of (myo)fibroblasts, see Cumming et al. (2009), Dickinson et al. (1994), Koppenol (2017), which is shown by Fig. 9d and e.

### Strain energy in the wound

Strain energy represents a measure of potential energy that is present in the tissue region. This potential energy results due to deformation and is computed by the integral over the computational region of half of the tensor inner product of the strain and stress tensors. Since it is believed that strain causes pain or itchy sensations to the patient, we use this quantity as a measure of pain that a patient has. Therefore, we are interested in the dynamics of this parameter, as well as the parameter yields understanding to the dynamics of the deformation and contraction of the wound.

**Fig. 10:**
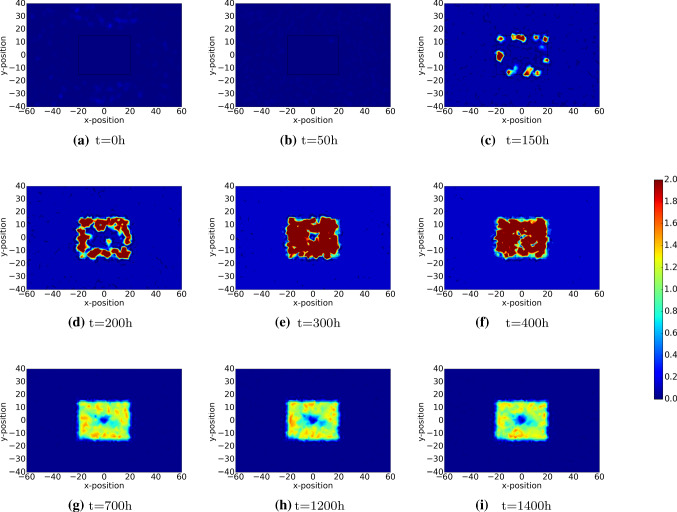
The plots show the strain energy distribution in the wound region, which is defined by $\int _{\Omega _w}\frac{1}{2}\underline{\underline{\sigma }}(\varvec{u}):\underline{\underline{\epsilon }}(\varvec{u})d\Omega_w$. The displacement of the extracellular matrix can be derived by solving Eq. 9 with Robin’s boundary condition. The parameter values used to solve the partial differential equations are taken from Table 1

The (myo)fibroblasts are responsible for the deformation of the tissue and hence these cells make the strain energy density increase locally and globally. Since initially the tissue is in mechanical equilibrium and since initially there are no (myo)fibroblasts present in the wound area, the strain energy density is zero in the wound initially. As time proceeds, the fibroblasts enter the wound region and myofibroblasts appear there, the strain energy locally increases in the wound area. The dynamics of the strain energy density field can be seen in Fig. 10. In Fig. 10c, the local increase due to appearance of (myo)fibroblasts has been visualized. After this stage, the strain energy density steadily increases in the wound region, and around 400h, it peaks and then decreases because of the apoptosis of the myofibroblasts, which removes the temporary deformation produced by them. However, since the permanent deformation caused by the myofibroblasts and the presenting of regular fibroblasts, the strain energy density of the wound will be positive constantly.

### Wound area reduction

We consider a two-dimensional representation of the wound, and we are interested in the contraction of the wound. To this extent, we compute the area of the wound over time and we compute the reduction of wound area over time by

29 $$\begin{aligned} r=\frac{A_\Omega }{A^0_\Omega }, \end{aligned}$$

where the area subject to deformation and reduction at any time is denoted by $A_\Omega$ and the initial wound area is represented by $A^0_\Omega$.

We use the shoelace theorem derived by Meister (1769) in 1769 to compute the wound area, since adjacent vertices on the wound boundary are connected and it results in a polygon. Suppose we have a polygon with *n* vertices, then the area is calculated by

30 $$\begin{aligned} A_{\Omega } \approx A_{\mathrm{SL}}=\frac{1}{2}\Vert \sum _{i=1}^{n}(x_iy_{i+1}-x_{i+1}y_i)\Vert , \end{aligned}$$

where $(x_i,y_i)$, $i=1,\ldots ,n$ is the coordinate of vertex *i* and $(x_{n+1},y_{n+1})=(x_1,y_1)$. Note that the vertices should be sorted in either a counter clockwise or clockwise direction.

**Fig. 11:**
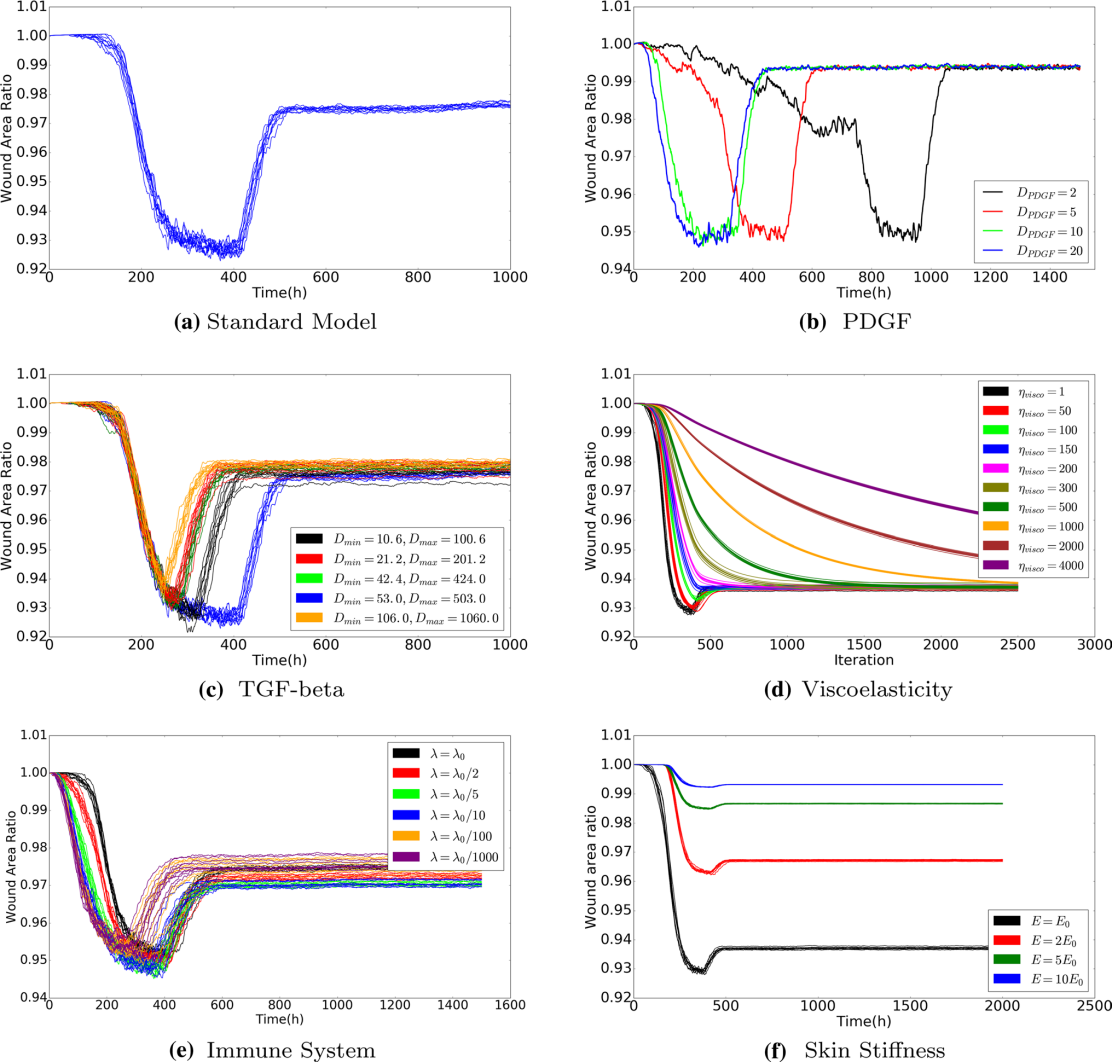
To investigate the influence of parameter values on wound healing, we use various values for the diffusion rate of PDGF and TGF-beta, the weight of viscoelasticity term for the force balance, the rate of immune cells random appearing, and the stiffness of the extracellular matrix in the model. The results are derived by solving the partial differential equations. The parameter values used to solve the partial differential equations are taken from Table 1, except the parameter under the sensitivity test

We plot the wound area as a function of time in Fig. 11, where various parameters have been varied in the different subplots. As an example, we consider Fig. 11a. The results in the other plots will be discussed in the next section. It can be seen that at the very early stages there is a slight increase in the wound area due to the pulling forces that are exerted by the fibroblasts that surround the wound region. After a while the fibroblasts migrate into the wound region and they start contracting the wound. This contraction is amplified after differentiation to myofibroblasts takes place. At the final stages of the simulations, the myofibroblasts and fibroblasts are subject to apoptosis and the myofibroblasts vanish, whereas the fibroblasts reach an equilibrium density, which is roughly equal to the density in the initial undamaged state (see also Fig. 5). At this stage the wound region retracts towards the initial state, but since permanent strains and deformations remain, see Fig. 10, it can be seen that there is a permanent reduction in the wound area, see Fig. 11a. Note that we have conducted multiple simulations in order to illustrate the impact of uncertainties that originate from the stochastic parts of the model (cell division, cell differentiation, cell death and cell migration).

### Sensitivity test of the model

Wound healing involves a cascade of biological processes in which many cellular phenomena take place and all changes in these cellular phenomena, either caused by diseases such as diabetes, or alternatively caused by genetic constitution or patients’ lifestyle can have significant impact on the evolution of the skin after wounding. Healing time, which is the time needed for the regeneration of collagen, but also contraction times and intensities are important parameters that are used to describe the physiological condition of the skin of the patient. Therefore, a sensitivity analysis of the model is indispensable. Since the complicated nature of the underlying model makes it impossible for us to find closed form expressions for the parameters of interest, we need to carry out computer simulations in which we change one or more of the input parameters. In Fig. 11, we show several simulation runs in which we use a basis set of parameter values from Table 1, and in which we change one of the input parameters. Since our model contains random processes for cell division, cell differentiation, cell appearance, and for cell migration, we conduct multiple simulations for some of the changes in the input parameters to see whether changes in the input parameter lead to significant changes in the behaviour of the solution.

The signalling molecules are crucially important for the sake of (long distance) intra-cellular communication. As a result of uncertainties from measurement and variation among patients and over time and tissue composition, diffusion coefficients vary unpredictably. Therefore, we vary the diffusion coefficient of these agents. In Fig. 11b, the diffusion coefficient of PDGF has been varied in several runs. Higher values of the diffusion coefficient of PDGF result into a faster transport of PDGF towards the immune cells, which are triggered earlier to migrate towards the wound region. Therefore, contraction takes place earlier as the PDGF diffusion coefficient increases, but the intensity of contraction both as a maximum and at the very long time range does not depend on the value of PDGF. Hence, the value of the PDGF diffusion coefficient does not effect the intensity of the contraction; it only impacts the length of the time-interval of the contraction process.

Skin tissue contains solid polymeric matter as well as liquid phases in the form of blood and fluidic substances in cells. Hence, viscous (friction) effects are of crucial importance to deal with. Viscous phenomena have a damping effect on the mechanical behaviour of the system. The forces that are exerted by the (myo)fibroblasts are damped by viscosity. This makes the deformation evolve more smoothly, and it makes the wound area evolve more smoothly over time. This can be seen in Fig. 11d, where large viscosities increase the damping component in the solution, and it can be seen that if the viscosity part in mechanics is very large, then the transition to the final contracted state proceeds even monotonic, without the presence of the minimum wound area at a finite time. Decreasing the viscosity makes the wound area exhibit a deeper minimum. Hence having a large viscosity, which corresponds to a higher degree of moisture, is beneficial for the patient at the short run, but does not change anything on the long run if therapy is not reconsidered. Since skin tends to become less moisture as skin ages, elderly people may suffer from a larger intermediate maximum degree of contraction (Gould et al. 2015).

**Fig. 12:**
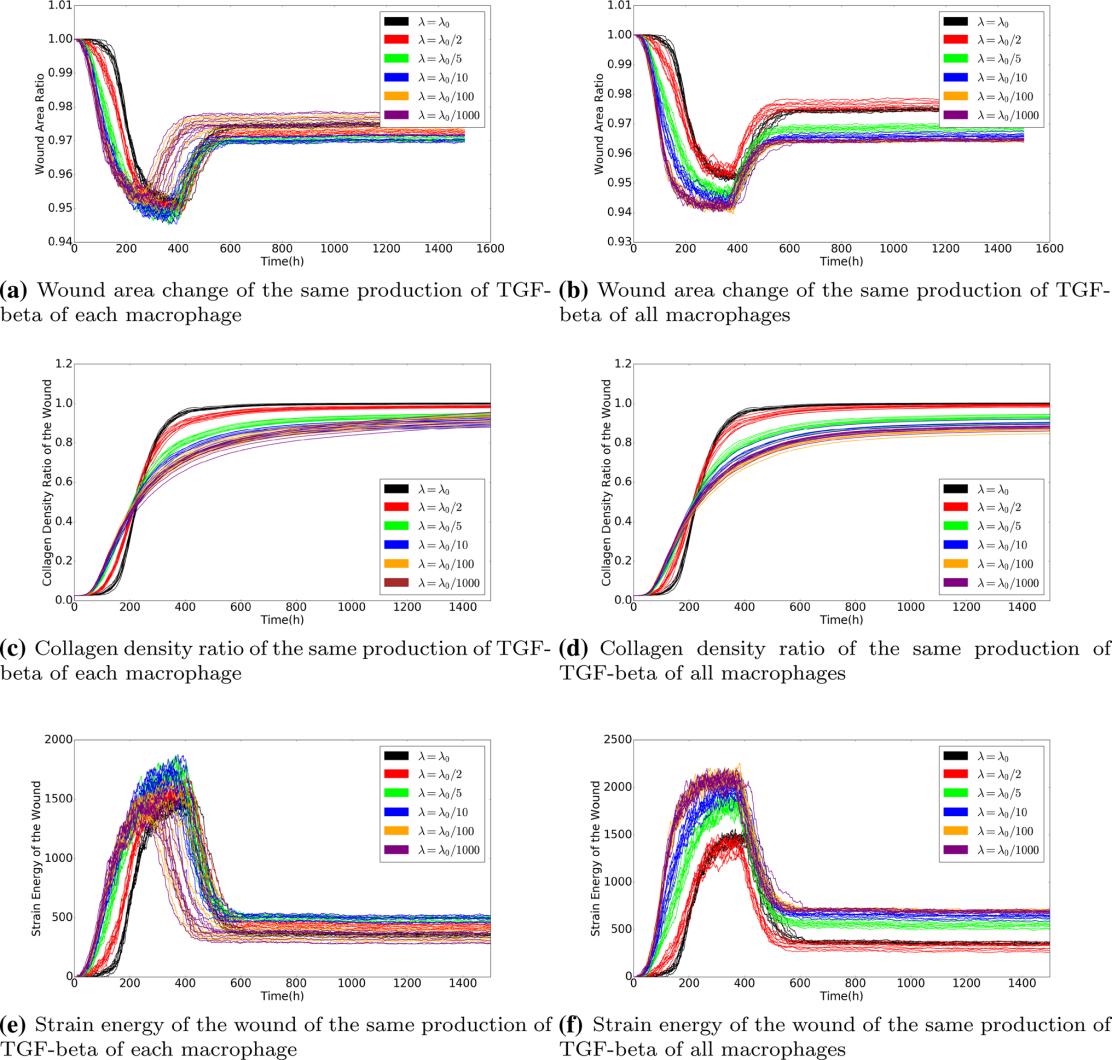
The left column shows the case when the production of TGF-beta of each immune cell is the same. The right column shows the case when the production of TGF-beta of all immune cells is the same. The results are derived by solving the partial differential equations. The parameter values used to solve the partial differential equations are taken from Table 1

We noticed that in Fig. 11e, the curves are not monotonic from the beginning of time. If the patient has a strong immune system, then more immune cells will appear on the edge between the wound and undamaged area. A large number of immune cells build up a ”cell wall” surrounding the wound and prevent fibroblasts entering the wound, despite the fact that the immune cells release large amount of TGF-beta to attract fibroblasts. On the other hand, if the patient has a weaker immune system, then the number of immune cells is not sufficient to promote fibroblasts entering, and hence the concentration of TGF-beta is not high enough to trigger efficient displacement of fibroblasts towards the wound. Hence, two different cases are investigated: the fixed production of TGF-beta of each immune cell and the fixed amount of production of TGF-beta of all the immune cells. In other words, we denote $N_0(t)$ and $N_1(t)$ as the number of immune cells at time *t* with different rate of immune cells random appearing, and $k_0$ and $k_1$ is the production of TGF-beta of each immune cell. Therefore, since we assume all the immune cells are identical, the two cases are: 1)$k_0=k_1$; 2)$N_0k_0=N_1k_1$, respectively. The wound area changes, the density of collagen ratio and the strain energy of wound are displaced in Fig. 12. We plot several curves that correspond to various strength of the immune system to have a better insight into the effect of the immune system on wound healing.

The stiffness of skin influences the magnitude of the displacement if a certain force is applied. Lower values of Young’s modulus (stiffness) increase the magnitude of the displacement for a given forcing (O’Leary et al. 2008). Hence, lower values of the stiffness make the skin contract more severely, which can be seen in Fig. 11f. Similar experimental results are observed in Wells (2013). However, the overall strain energy does not necessarily increase since the strain energy is proportional to the skin stiffness. Skin stiffness can be related to age and other genetic patterns of the patient. Age, however, is a complicated, but important, parameter since it may incur simultaneous changes of several parameters (Thomas 2001).

Based on the simulations that we have done so far, the results seem to reproduce most of the measured trends and can be explained by an intuitive point of view. In the next section, we will present the results from Monte Carlo simulations in which a Bayesian parameter variation study has been carried out.

In every subplot of Fig. 12, the probability rate for the appearance of the immune cells (macrophages) has been varied. Comparing the curves in each row of the subplots, it can be concluded that the collagen density ratio cannot indicate the degree of the wound contractions, but the wound strain energy dynamics follows the same tendency of wound area change. This conclusion can also be derived from the correlation in Monte Carlo simulations, which will be discussed in the next section. In other words, the correlation between the wound strain energy and the wound area is relatively linear and significant.

**Fig. 13:**
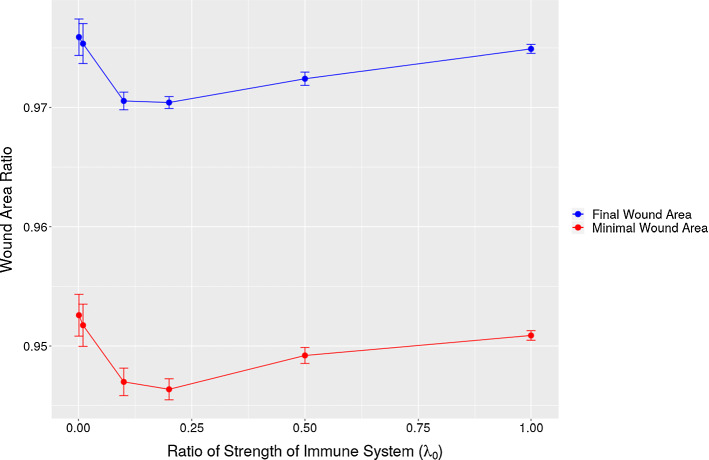
Minimal and final wound area ratio with respect to various probability rates of immune cells appearance. The blue curves and points stand for the final wound area, and the red curves and points present the minimal wound area. The parameter values used to solve the partial differential equations are taken from Table 1

More importantly, it can be seen that if this probability rate of macrophages appearance is large, then the count of immune cells is large. This implies that the concentration of TGF-beta is larger and hence the (myo)fibroblasts arrive into the wound site at earlier stages and in larger numbers. Therefore, at larger probability rates of macrophage appearance, the wound area decreases faster and earlier and to lower values, which implies that the maximal degree of contraction is larger. In fact, we expected that the permanent (plastic) deformations are also larger and hence the final degree of contraction is also larger, which corresponds to a smaller final wound area, since the counts of fibroblasts and hence also myofibroblasts are larger for larger values of the probability rate. However, the results in either Fig. 12a or b are not monotonic, which results from that larger number of immune cells will build up a barrier on the boundary of the wound, preventing the fibroblasts entering the wound to cure it. Therefore, above some threshold of probability rates of macrophage appearance, the larger the rates, the less the contraction; when the rates are below the threshold, the less the rates, the less the contraction. In other words, there is an (non-favourable) optimum for the degree of contraction with respect to the probability rate of macrophage appearance; see Fig. 13. From the patient’s point of view, a lower value of lambda decreases the onset of wound healing and regeneration of collagen, which hence delays wound healing, however, wound contraction is inhibited by lower lambda values. Hence, a slower immune reaction could be beneficial to the patient in this sense. A similar conclusion was drawn in Koppenol (2017), Larouche et al. (2018).

**Fig. 14:**
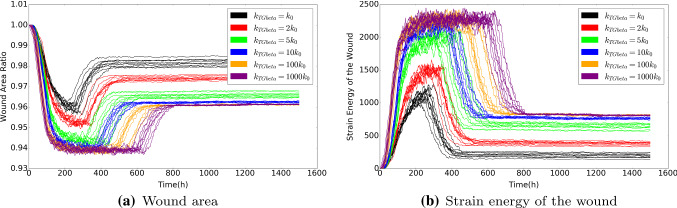
The area and the strain energy of the wound with various values of the production rate of TGF-beta. The parameter values used to solve the partial differential equations are taken from Table 1

Besides the barrier built up by large number of immune cells, the main effect of the probability rate of immune cells appearance is the production of TGF-beta. Hence, we conduct another series of simulations where the TGF-beta production rate (denoted by $k_{\mathrm{TGF}}$) per macrophage has been varied. A low value of $k_{\mathrm{TGF}}$ corresponds to a relatively weak immune system. In this case, we avoid the scenario of having a wall of immune cells that prevent fibroblasts to enter the damaged region. Figure 14 shows the wound area and the strain energy of the wound against time. The results in both subplots are monotonic, which indicates that the more production of TGF-beta, the more contraction and the larger strain energy of the wound, as well as shorter healing time, since the fibroblasts proliferate more and differentiate more into myofibroblasts and get stronger signals to move towards the wound. In other words, in our model, TGF-beta is the key factor why the strength of the immune system influences the healing process.

## Monte Carlo simulations

As we mentioned earlier in Table 1, the parameter values are mostly selected based on the sensitivity of the model. Additionally, due to the limited literature sources of *in vivo* data for these parameters, and the discrepancy between the values collected by different measurements in the laboratory (Evans et al. 2013; Liang et al. 2010; Solon et al. 2007; Wakhlu et al. 2017), Monte Carlo simulations will be beneficial to probe the hidden correlation between the parameters. The input parameters (see Table 2) represent different characteristics of patients, for instance, age, strength of immune system, etc. (Thomas 2001). We are interested in the contractions, which is indicated by the volume change. For each category, we recorded the final (equilibrium), minimal area (most intensified contraction) and the scar area at the 4th day after wounding.

**Table 2:** Distributions of the input parameters in Monte Carlo simulations

Parameter	Description	Distributions
Input parameters		
$E\_s$	Substrate elasticity	Log normal $(\log 50, 0.1)$
$\lambda _d$	Division rate of regular fibroblasts related to strain energy density	Uniform (1.5, 2.5)
$\lambda _a$	Apoptosis rate of myofibroblasts	normal(10, 0.1)
$\lambda _{\mathrm{immune}\_\mathrm{random}}$	Rate of Point Poisson Process of macrophages random appearing on the edge between the wound and undamaged skin	log normal$(\log 0.04,10^{-5})$

Responses/outputs		
Parameter	Description	Mean (SD)
*n*	The time when the model reaches equilibrium	626.4(38.46908)
$n\_\hbox{min}$	The time when the wound has minimal volume	380.1(27.64454)
$\hbox{Area}\_\hbox{final}$	The equilibrium wound area	1142(4.658205)
$\hbox{Area}\_\hbox{min}$	The minimal wound area	1090(8.324712)
$\hbox{Area}\_4\,\hbox{days}$	The wound area at 4th day after wounding	1198(1.448219)
$\hbox{rho}\_c\_\hbox{hat}\_\hbox{final}$	The ratio of average density of collagen when the model reaches equilibrium	0.9950(0.003062998)
$\hbox{rho}\_c\_\hbox{hat}\_\hbox{min}$	The ratio of average density of collagen when the wound area is minimal	0.9336(0.02632869)

We collected 1210 observations via the simulations. The description of the data is shown in Table 2. Regarding the effect from inputs on the outputs, the stiffness of the skin has a significant correlation with the minimal and final area (0.97 and 0.96, respectively), as well as the ratio of the average density of collagen over the wound (0.39). The death rate of myofibroblasts does have a relatively less significant correlation and weak correlation (0.048) with the ratio of the average density of collagen over the wound.

However, the responses show more significant correlations. With respect to the final area of the wound, the final area of the wound has a significant correlation with many other outputs. In particular, it has an almost linear correlation with the minimal wound area, whereas the correlation is 0.17 with the wound area at the 4th day after wounding. Therefore, it would be beneficial for physicians to predict the final healed wound with these data known.

Further we investigate the correlation between the final density of collagen and several other parameters. Even though it shows mostly a significant correlation with other output, all the correlations are not relatively high—the absolute value are all below 0.4, except the one with the time when the model reaches its equilibrium. Thus, regarding the wound contractions, the final density ratio cannot provide much information about the degree of the wound contractions.

**Fig. 15:**
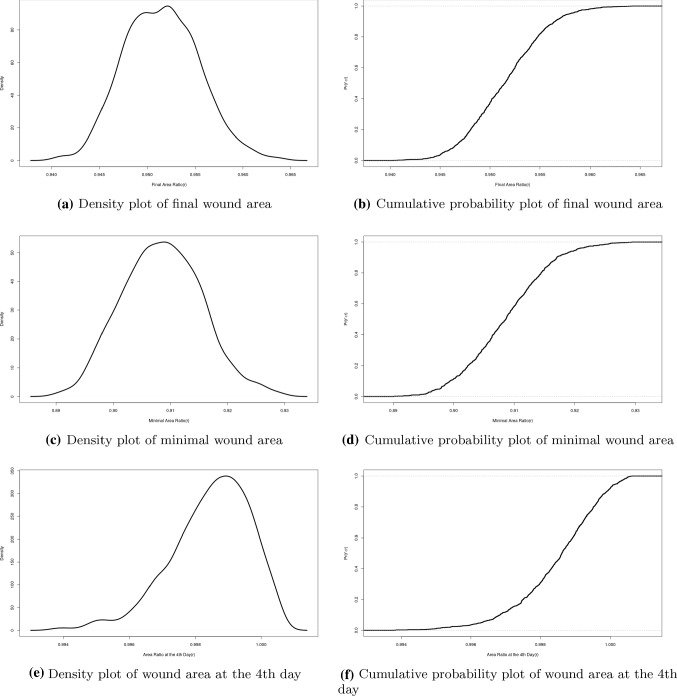
Left column shows the density plots of the wound area at different time. Right column shows the probability function plots of the wound area at different time. The parameter values used to solve the partial differential equations are taken from Table 1, and the random variable value are from Table 2

We investigate the statistical distributions of the wound area at multiple times. This gives an indication of the severeness of contractions that patients will develop. Hereby, we use the ratio described in Eq. (29). The density plots and the cumulative probability function plots are shown in Fig. 15.

**Fig. 16:**
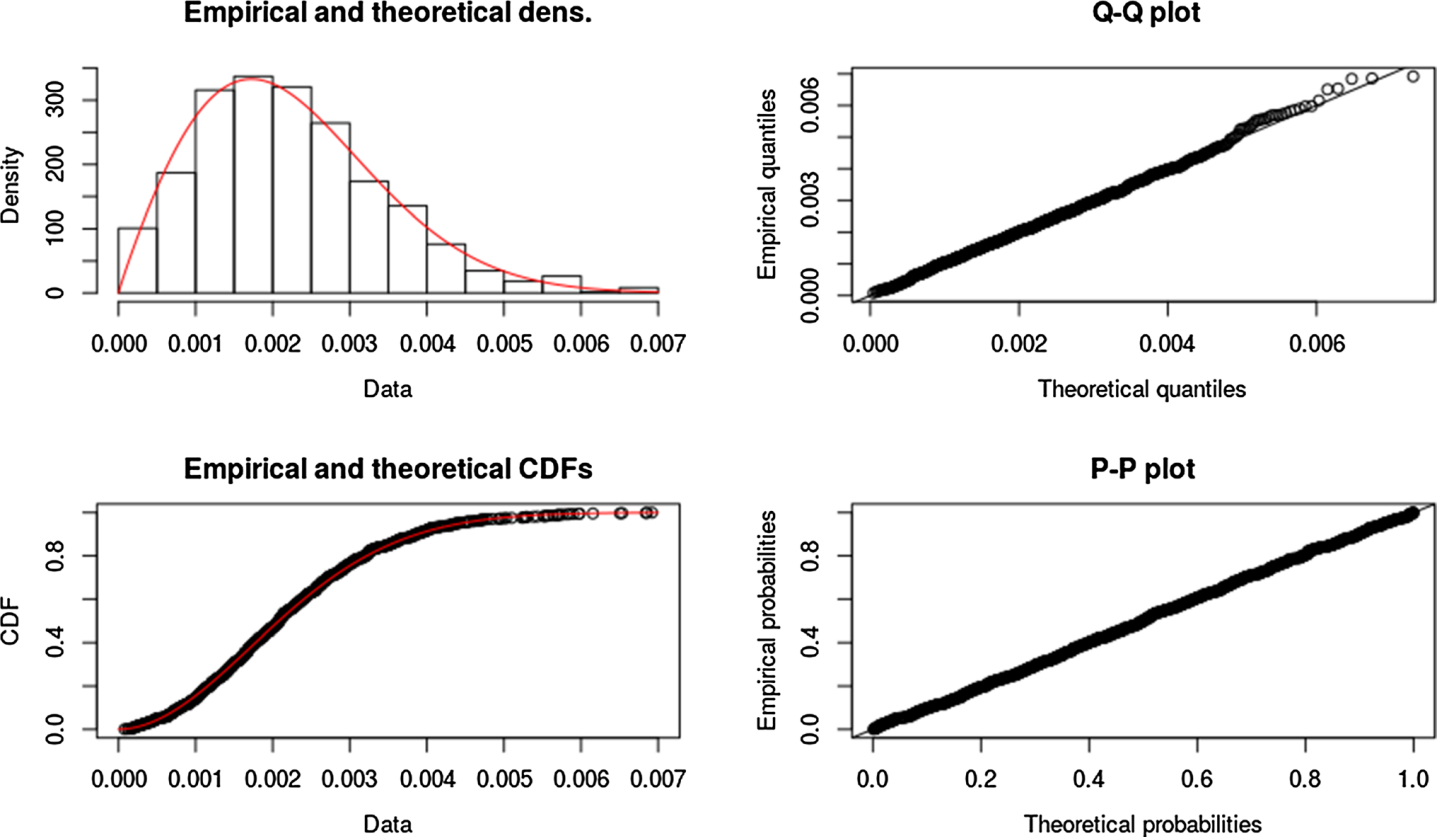
The diagonal plots of Weibull distribution fitting the reversed data of the wound area at the 4th day

The curves in Fig. 15a and c show the Gaussian shape. However, according to the Shapiro normality tests (Shapiro and Wilk 1965), neither of them come from a normal distribution. For the data of the wound area at the 4th day, we use Weibull model to fit the reversed data, that is, we investigate the maximum of the data plus a small positive number subtracting every data, since the Weibull distribution requires all the positive data. We used 2-parameter Weibull distribution. The estimates of the parameters are shown in Table 3, and the diagonalized plots are displayed in Fig. 16. It can be concluded that Weibull distribution fits the reversed data quite well.

**Table 3:** Weibull distribution to fit the reversed data of wound area at the 4th day

	Estimate	Standard error
Shape	1.92876	$4.42490\times 10^{-2}$
Scale	$2.51742\times 10^{-3}$	$1.49013\times 10^{-5}$
Log-likelihood: 6492.219	$\hbox{AIC}=-12980.44$	

From Fig. 15b, patients have 0.627 probability to recover at most 95% of the original wound volume. Figure 15d indicates that for the largest contraction in small-scaled wound, the possibility that patients lose 9% of the volume is 0.585.

**Fig. 17:**
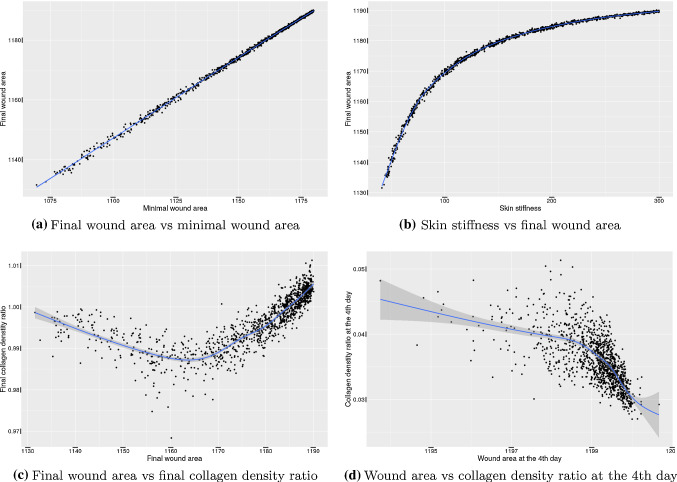
The plots show the correlation plot of two parameters from the Monte Carlo simulations. The curve in each plot is the smoothed line with the confidential interval. In each subplot, the *p*-value of the correlation is less than 0.001. The parameter values used to solve the partial differential equations are taken from Table 1, and the random variable value are from Table 2

In the first batch of the Monte Carlo simulations, we have already concluded that the stiffness plays an important role in influencing the outcomes of the wound healing, in spite of a relatively small range that was used for the skin stiffness $E_s$. In the second round, we keep every distribution of the input the same as in the previous batch except for the stiffness. Herewith, we use the uniform distribution ranged from 23.90 to 300.00 from Liang et al. (2010). Additionally, the collagen density ratio at three different time points (i.e. when the wound area reaches equilibrium, minimal and at the 4th day) has been recorded as well as outcome. We plot out some significant and interesting correlation results in Fig. 17. Mostly, the correlation relations do not vary a lot. Furthermore, the final area and the minimal area has a correlation of 1.0, and the correlation between the wound area at the 4th day and the final/minimal wound area is 0.53, which is much larger than the one before. Between the final/minimal wound area and the collagen density ratio at the corresponding time point, it shows a non-monotonic “valley” shape, which matches the results in the sensitivity test before, that is, the collagen density ratio cannot indicate how severe the wound contraction is. However, when the new collagen is still regenerated from null, there exists a strongly negative linear correlation ($-\,0.64$) between the wound area and the collagen ratio density, which can be seen from the data at the 4th day post wounding. Next to it, we noticed that the distribution of final/minimal wound area is more like an inverse Weibull distribution than a Gaussian distribution, which illustrates how significantly important the skin stiffness is to the wound healing, with respect to contraction.

## Discussions and conclusion

In this manuscript, we mainly implement the semi-stochastic model to mimic the wound healing processes, and in particular the chained processes of inflammation and subsequent collagen regeneration. In the model, the displacements of cells, the dynamics of signalling molecules and wound area contractions over time are all described by PDEs. The model seems to reproduce the trends and biological observations reasonably well.

As it is seen from the results, macrophages play an important role in the early stage and they are the first arriving in the wound. In the meanwhile, TGF-beta is secreted by macrophages and this chemical attracts regular fibroblasts, as well as trigger the differentiation from regular fibroblasts to myofibroblasts. The number of macrophages and myofibroblasts follows the same pattern, i.e. increasing first then drop down to zero, whereas the number of regular fibroblasts will drop down to a certain level.

Signalling molecules are crucial for the chemotaxis of cells. Starting from higher concentration in the wound area and lower values in healthy tissues, PDF declined to zero over the whole observed domain in the end. Since macrophages are the main source of TGF-beta, the dynamics of the concentration is the same as the number of macrophages.

The wound contractions are illustrated by the area change of the wound. Initially, fibroblasts are outside of the wound; hence, the forces they exert first enlarge the wound area a little. Due to the high concentration of TGF-beta, they gradually move into the wound and cause the deformation, that is, the reduction of wound area and accumulation of strain energy over the wound region. As the number of fibroblasts eventually stabilizes, the area of the wound and strain energy also tend to reach a stable equilibrium..

Under practical circumstances, the fibrin and collagen will affect the activities of the cells. To be more specific, they provide the guidance of the direction of the displacements of the cells. In addition, the bundles of these tissues will increase the strain of the wound and yield to the problematic complications after the wound healing. We have incorporated the tissues and related signalling molecules like tissue plasminogen activator (tPA) to improve the model.

To some extent, the model matches certain important time points with clinical data, for example, the maximal concentration of TGF-beta occurs around 100 h after wounding (Cumming et al. 2009; Dallon et al. 2001), the regular fibroblasts started arriving in the wound region in two to 5 days after the injury and the number of (myo)fibroblasts peaks at one to 2 weeks post-wounding (De la Torre and Sholar 2006), whereas in our model, (myo)fibroblasts appear in the wound around 4th days after wounding and the peak of the sum of myofibroblasts and regular fibroblasts appears 12 days after wounding. In addition, after the sensitivity tests of the model, the conclusions drawn are matched with other experimental observations like Larouche et al. (2018); O’Leary et al. (2008) and Wells (2013) etc.

According to the sensitivity tests, we found out that the most factors have a monotonic influence on the wound contractions except the probability rate of immune cells appearance. Since the probability rate of immune cells appearance is related to the number of immune cells and subsequently decides the production of TGF-beta, there exists an optimal possibility rate of immune cells appearance. This is attributed to the fact that as too few immune cells do not release enough TGF-beta, whereas too many immune cells build up a barrier on the edge of the wound preventing the fibroblasts entering the wound region. Therefore, below some threshold, a weaker immune system will extend the healing time but also, favourably, result into a less contracted wound.

The model is the basis to do Monte Carlo simulations, which are meant to figure out the effect of certain components on the healing process. We found out that the stiffness has a significant influence on wound healing regarding the contractions, which is also verified by *in vivo* and experimental observations. The distribution of the equilibrium and the minimal area is highly dependent on the distribution of skin stiffness, and there is a (nearly) significantly linear correlation between them. According to the significant correlations between the area at the 4th days after wounding and the equilibrium and minimal area, the area at the 4th day, is supposed to provide some information about the healing process afterwards. However, the relation between the wound area and the corresponding collagen density ratio is not monotonic, while for the 4th day the collagen density ratio can be used to indicate the degree of the wound contractions.

Since the cell-based model is mainly for the small-scale wound, the area reduction ratio is relatively small. The probability that patients will end up with 5% contraction or less is about 0.627, and have the worst contraction with losing 9% of the volume is around 0.585, with a small range of skin stiffness.

We bear in mind that the model is currently only two dimensions, which has the benefits of efficient computations for a large number of simulations. In particular, obtaining results from Monte Carlo simulations is more efficient. On the other hand, we are working on extending the model into three dimensions, which is more realistic but more expensive in the computational perspective.
